# Uncovering pan-cancer signatures of chemoresistance

**DOI:** 10.1186/s13073-026-01763-2

**Published:** 2026-09-16

**Authors:** Mohammed Inayatullah, Engin Demirdizen, Zachery Keepers, Catarina Mendes Correia, Seyyed Morteza Hashemi, Kaushlendra Tripathi, Souren Sadhukhan, Ishita Bardhan, Arun Mahesh, Feyruz V. Rassool, Mikkel Green Terp, Hem D. Shukla, Vijay K. Tiwari

**Affiliations:** 1 Institute of Molecular Medicine, University of Southern Denmark, Campusvej 55, Odense M, DK-5230 Denmark; 2 Division of Translational Radiation Sciences, Department of Radiation Oncology, University of Maryland School of Medicine, Baltimore, MD 21201 USA; 3 University of Maryland Marlene and Stewart Greenebaum Comprehensive Cancer Center, Baltimore, MD 21201 USA; 4 Danish Institute for Advanced Study (DIAS), University of Southern Denmark, Campusvej 55, Odense M, DK-5230 Denmark; 5 Department of Clinical Genetics, Odense University Hospital, Odense C, DK-5000, Denmark; 6 Mohamed Bin Zayed University of Artificial Intelligence, Abu Dhabi, UAE; 7 Wellcome-Wolfson Institute for Experimental Medicine, School of Medicine, Dentistry & Biomedical Science, Queens University Belfast, Belfast, BT9 7BL UK

**Keywords:** Chemoresistance, Cancer, Single-cell heterogeneity, Gene expression, Drug discovery

## Abstract

**Background:**

Chemotherapy resistance remains a formidable challenge in cancer treatment, driving high mortality rates worldwide. Despite significant advancements, it remains unclear whether conserved molecular programs underpin therapy resistance across cancer types.

**Methods:**

Here, we integrate single-cell RNA sequencing, spatial transcriptomics, regulatory network modeling, transcription factor binding data, and pharmacologic perturbation across multiple cancer types to define a conserved, proliferative chemoresistant tumor state.

**Results:**

Contrary to the prevailing notion that resistance arises from quiescent or EMT-like phenotypes, we find that resistant tumor cells display elevated G2/M and S-phase activity, enriched expression of E2F and MYC target genes, and activation of DNA repair and PI3K/AKT signaling pathways. We identify the transcription factor MYC as a central regulator of the resistant state, with progressive activation along the resistance trajectory and focal expression in resistant epithelial niches. A novel MYC target, SRM (Spermidine Synthase), emerges as a conserved effector of resistance, promoting polyamine biosynthesis critical for chromatin stability and metabolic resilience. SRM expression correlates with MYC binding and predicts poor patient survival. Functional validation in cell lines, patient-derived organoids and mouse models demonstrate that pharmacologic inhibition of MYC, SRM, or WNT signalling restores chemotherapy sensitivity, suppresses resistance-associated pathways, and reactivates apoptosis. Spatial and survival analyses confirm the clinical relevance of the MYC–SRM axis, establishing it as a druggable module in treatment-refractory cancers.

**Conclusions:**

To our knowledge, this is the first comprehensive study that redefines chemoresistance as a proliferative, MYC-driven state and uncover SRM as a tractable vulnerability, offering new avenues for therapeutic intervention across diverse epithelial malignancies.

**Supplementary Information:**

The online version contains supplementary material available at https://doi.org/10.1186/s13073-026-01763-2.

## Background

Chemotherapy is a cornerstone of cancer treatment, but tumors frequently develop resistance against it, rendering the therapy ineffective. Chemoresistance is a major hurdle, contributing up to 90% of cancer-related deaths worldwide and serving as a critical barrier to improving clinical outcomes, particularly in aggressive cancers. Overcoming therapy resistance is essential for enhancing survival rates and achieving more effective cancer management strategies [1, 2]. Despite substantial advances in anticancer drug development, intrinsic and acquired treatment resistance continue to limit the durability of therapeutic responses and remain major causes of treatment failure across cancer types [1–3]. This underscores the urgent need for more effective therapeutic strategies to overcome therapy resistance in cancer.

Resistance to anticancer drugs is driven by a complex set of factors, including genetic mutations, epigenetic alterations, enhanced drug efflux, and so on. Additionally, tumor heterogeneity, the diversity of cell populations within the tumor, plays a pivotal role in treatment failure. This cellular complexity is a major obstacle in effectively treating cancer, as cellular subpopulations can variably respond to therapies, promoting survival and resistance [4]. The emergence of single-cell technology has provided great potential to study molecular changes at the single-cell resolution, allowing the discovery of novel genomic, epigenomic, and transcriptomic signatures [5, 6]. However, previous studies of therapy resistance have largely targeted single subtypes within the spectrum of a given disease and no efforts have been made to identify universal mechanisms and therapeutic targets within that could apply across cancers. We hypothesize that core molecular circuitry underlying chemoresistance might be shared across cancers, and identifying encoded common molecular pathways will advance our understanding and therapeutic strategies to overcome these barriers.

As therapy resistance presents a key challenge across diverse cancer types, a pan-cancer investigation offers significant advantages. Firstly, it can reveal conserved molecular pathways crucial for therapy resistance, facilitating the identification of therapeutic targets detectable across a spectrum of cancers. Moreover, the incorporation of multiple cancer types enhances statistical power, enabling the detection of subtle yet biologically significant differences in gene expression and cell phenotype linked to therapy resistance. The insights gained are inherently generalizable, ensuring relevance across diverse patient populations, consequently increasing clinical importance. Ultimately, pan-cancer studies play a key role in identification of common targets, which underlie in therapy resistance, overcoming the complication associated with different cancers. Here using single-cell and spatial transcriptome of aggressive tumors from four major cancer types, including breast [7, 8], lung [9, 10], ovary [11], and pancreas [12, 13], in combination with state-of-the-art computational approaches and functional analyses, we have uncovered cellular diversity and underlying transcriptional programs linked to chemoresistance and identified biomarkers to stratify chemoresistant patients and therapeutic targeting across cancers.

## Methods

### scRNA-seq patient cohorts, data processing and integration

Single-cell RNA sequencing data from chemoresistant and chemosensitive tumors were obtained from previously published studies comprising 29 tumors across four major cancer types: i.e. Breast [7, 8], Lung [9, 10], Ovary [11], and Pancreas [12, 13] cancers.

Patients represented in these datasets had received platinum-, FOLFIRINOX-, and/or taxane-based chemotherapy, either alone or in combination, as reported in the original studies. Following quality-control filtering, a total of 126,724 cells were included in the integrated analysis, comprising 60,171 cells from chemoresistant tumors and 66,553 cells from chemosensitive tumors. Detailed information on the individual datasets, including sample characteristics, treatment and response status, original study, and accession information, is provided (Additional file 2: Table S1).

The preprocessed of the dataset was performed and low-quality cells were removed from the analysis and high-quality cells considered for further analysis. The possible batch effects across multiple samples were corrected, and scRNA-seq datasets were integrated using the canonical correlation analysis (CCA) method in Seurat. The effectiveness of batch correction was assessed by ensuring that cells were clustered according to distinct cell types rather than patient samples.

### Cell-type annotation and differential analysis

Cell-type identities were assigned using canonical marker genes obtained from CellMarker 2.0 (Additional file 2: Table S2). To accommodate the pan-cancer nature of the dataset, tissue-specific marker references corresponding to breast, lung, ovarian, and pancreatic tissues were retrieved from CellMarker 2.0 and integrated with established lineage markers reported in the literature. Cluster-specific marker genes were first identified using differential expression analysis and subsequently matched against the curated marker database [14]. To identify malignant cells within the integrated scRNA-seq datasets, we used the inferCNV (v1.3.3) package with default settings [15]. The differentially expressed gene between chemoresistant and chemosensitive for each cell types were identified using DESeq2 [16] in Seurat package. Differentially expressed genes to each cell types were further mapped Molecular Signatures Database (MSigDB) [17] using clusterProfiler pathway analysis tool [18] to identify activated and downregulated functional pathways in chemoresistant tumors.

### Cell-cycle analysis

Cell-cycle states were inferred using the CellCycleScoring() function implemented in Seurat (v5). Briefly, each cell was scored using canonical S-phase and G2/M-phase signatures of 97 genes (Additional file 2: Table S3) originally reported by Tirosh et al., 2016 [19]. The algorithm calculates expression-based scores for S-phase and G2/M-phase marker genes and assigns each cell to G1, S, or G2/M phase according to the dominant cell-cycle program. Cells exhibiting higher expression of S-phase genes were classified as S phase, whereas cells enriched for G2/M-associated genes were assigned to G2/M phase. Cells lacking enrichment for either program were classified as G1 phase. The resulting cell-cycle assignments were subsequently used to compare proliferative states between resistant and sensitive tumor populations.

### Cancer cell-state analysis

Functional cancer cell states were inferred using curated gene signatures obtained from the CancerSEA database, a single-cell functional atlas of cancer that systematically annotates genes associated with distinct cellular phenotypes across multiple cancer types [20]. CancerSEA signatures encompass a broad spectrum of cancer-associated states, including proliferation, cell cycle, DNA damage, DNA repair, apoptosis, epithelial–mesenchymal transition (EMT), stemness, invasion, metastasis, angiogenesis, differentiation, hypoxia, inflammation, and quiescence.

For each malignant cell, enrichment scores for individual CancerSEA signatures were calculated using expression-based gene-set scoring. State scores were subsequently aggregated and compared between chemoresistant and chemosensitive tumor populations to identify biological processes associated with resistance.

### Spatial transcriptomic datasets

Spatial transcriptomic datasets were obtained from publicly available studies of breast cancer [21], pancreatic cancer [22], ovarian cancer [23], and lung cancer (10x Genomics Visium dataset). The breast, pancreatic, and ovarian cohorts represented clinically aggressive tumors, including treatment-resistant, residual, recurrent, or advanced disease samples, as described in the respective original studies. Patient recruitment, tissue collection, sample processing, and clinical characterization were performed as reported in the original studies. Spatial expression matrices and associated histological images were obtained from the corresponding studies/public repositories. The same spatial transcriptomic datasets were used throughout this study to assess the spatial distribution of the chemoresistance signature and *SRM* and *MYC* expression within tumor and stromal compartments.

### PANCANCER resistant signature identification and resistant state trajectory construction

A chemoresistance trajectory was constructed within malignant cells classified as chemoresistant to capture intra-resistant heterogeneity. A chemoresistance gene signature, derived from differential expression analysis between resistant and sensitive malignant cells, was used to compute a resistance score for each cell. Based on this score, resistant malignant cells were stratified into Low, Medium, and High resistance states. Cells were then ordered along a continuous trajectory according to their resistance score, enabling the reconstruction of a resistance progression axis. This approach allowed the identification of transcriptional and regulatory changes associated with incremental increases in chemoresistance.

### Resistant signature validation in bulk RNA-seq

To validate single-cell findings in independent patient cohorts, publicly available bulk RNA-seq datasets from chemotherapy-treated patients with lung, breast, pancreatic, and ovarian cancers were analyzed using the CTR-DB 2.0 database [24] (http://ctrdb.cloudna.cn/). Chemotherapy-treated patient cohorts with annotated response information (sensitive vs. resistant) were selected The cohorts used for *SRM* expression analysis comprised breast (*n* = 113), lung (*n* = 73), pancreatic (*n* = 107), and ovarian (*n* = 58) cancer samples. Independent cohorts used for *MYC* expression analysis comprised breast (*n* = 38), lung (*n* = 73), pancreatic (*n* = 66), and ovarian (*n* = 58) cancer samples. Sample collection, patient enrollment, treatment, and response assessment were performed as described in the respective original studies. Expression of the chemoresistance gene signature and key biomarkers, including *SRM* and *MYC*, was compared between resistant and sensitive groups using the statistical framework implemented in CTR-DB. Differential expression analysis between groups was performed using built-in CTR-DB statistical frameworks, and statistical significance was assessed using appropriate tests (e.g., Wilcoxon rank-sum test). This analysis enabled validation of single-cell–derived resistance programs and key drivers in large, clinically annotated bulk transcriptomic datasets.

### Resistant signature validation in across normal, primary, and metastatic tissues

To further validate the clinical relevance of the identified chemoresistance signature and key biomarkers, we analyzed gene expression across healthy normal, primary tumor, and metastatic tissues using the TNMplot platform [25] (https://tnmplot.com/analysis/) for lung (*n* = 2,216), breast (*n* = 7,805), pancreatic (*n* = 373), and ovarian (*n* = 814) cancers. Expression levels of the chemoresistance gene signature, as well as *SRM* and *MYC*, were extracted from publicly available bulk RNA-seq datasets integrated within TNMplot. Gene signature scores were calculated as the average expression of signature genes per sample. Comparative analyses between normal, primary, and metastatic groups were performed using the statistical framework provided by TNMplot (e.g., Kruskal–Wallis and Mann–Whitney tests). This analysis enabled assessment of the association between chemoresistance programs and tumor progression, particularly their enrichment in metastatic disease.

### Resistant signature spatial analysis

Spatial transcriptomic analysis was performed on 10x Genomics Visium datasets from lung, breast, pancreatic, and ovarian cancers using the Seurat (v4) R package. Raw Visium data were processed using the standard Seurat workflow, including quality control, normalization (SCTransform), and dimensionality reduction. Tumor microenvironment including tumor, stromal and immune compartment were annotated using Spatial Cellular Estimator for Tumors (SpaCET) tool [26], which estimates cancer cell abundance by integrating a gene pattern dictionary of copy number alterations and expression changes in common malignancies. A chemoresistance gene signature score was computed for each spot using Seurat’s AddModuleScore function. In parallel, expression levels of key biomarkers *SRM* and *MYC* were quantified across spatial locations. Spatial feature plots were generated to visualize the distribution of chemoresistance scores and gene expression within the tumor microenvironment. Comparative analyses were performed to assess enrichment of chemoresistance programs in specific spatial regions (e.g., tumor core vs. stromal compartment). This approach enabled spatial mapping of chemoresistance-associated transcriptional programs and their relationship to tumor architecture across multiple cancer types.

### Transcription factor (TF) regulatory network

Transcription factor (TF) regulatory network analysis was performed on low, medium and high resistant state malignant cells using CellOracle (v. 0.10.15) [27] following tutorials from https://morris-lab.github.io/CellOracle.documentation/. A prior TF–target interaction network derived from motif databases (JASPAR) [28] was integrated with single-cell expression data to infer context-specific gene regulatory networks. TF activity scores were calculated for each cell and compared across resistance states to identify key regulators.

### Resistant signature survival analysis

Kaplan–Meier (KM) survival analysis was performed to evaluate the clinical relevance of identified gene signatures across multiple cancer types. Overall/Relapse-free survival survival associations were assessed using the KM Plotter online tool [29] (https://kmplot.com/analysis/) for lung, breast, pancreatic, and ovarian cancers cohorts. Patients were stratified into high and low expression groups based on the median expression of the gene or resistant gene signature or SRM alone. Survival curves were generated using the Kaplan–Meier method, and statistical significance was evaluated using the log-rank test. Hazard ratios (HR) with 95% confidence intervals were calculated to quantify the association between gene expression and patient survival outcomes.

### ChIP-seq analysis

The *MYC* ChIP-seq datasets of six *human* cancer cell lines (MCF7, BT549, SW1271, UACC812, MDA-MB-468, and A549) were obtained from ChIP-Atlas [30], which processes public datasets using MACS2 [31] with the default Chip-seq analysis pipeline with hg19 genome version. Peaks were identified based on enrichment over background using a Poisson model, with statistical significance assessed by *p*-values and false discovery rate (FDR)-adjusted q-values. Peaks were filtered using predefined stringency thresholds (e.g., q < 1e − 5 or q < 1e−10), and peak scores were defined as −log10(q-value). Fold enrichment over input and peak summit positions were used to quantify binding intensity. The analyzed MYC chip-seq dataset and bound sites were visualize using UCSC genome browser.

### Machine-learning based classification of cancer cell lines

To identify representative cellular models of chemoresistance, transcriptomic profiles of cancer cell lines were obtained from the Cancer Cell Line Encyclopedia (CCLE) [32] available in DepMap [33]. We used the 100-gene chemoresistance signature identified from our integrated single-cell analysis of resistant and sensitive tumors (Additional file 2: Table S4) as the feature set for classification. For each cell line, normalized expression values of the chemoresistance signature genes were extracted and used as model input features. Cell lines were classified into resistant-like or sensitive-like groups based on their expression patterns.

Multiple supervised machine-learning algorithms were evaluated, including Decision Tree, Logistic Regression, Support Vector Machine (SVM), Multi-Layer Perceptron (MLP) with one hidden layer, and MLP with two hidden layers. The dataset was randomly divided into 80% training data and 20% testing data. Models were trained using the training dataset and subsequently evaluated on the independent testing dataset. Classification performance was assessed using prediction accuracy.

To independently validate the biological relevance of the predictions, drug-response profiles for cisplatin and paclitaxel were obtained from the Genomics of Drug Sensitivity in Cancer (GDSC) database [34]. Cell lines predicted as resistant-like displayed lower sensitivity to both chemotherapeutic agents compared with sensitive-like cell lines. Based on these analyses, MCF7 (breast cancer), PATU8988T (pancreatic cancer), and A2780/CP70 (ovarian cancer) were selected as representative chemoresistant models for downstream functional validation.

### Cell culture

PATU8988T PDAC cell line and MCF7 breast cancer cell line were maintained in DMEM (Dulbecco’s modified Eagle’s medium, Gibco) supplemented with 10% FBS. Cells were maintained in DMEM (Dulbecco’s modified Eagle’s medium, Thermo #D5796) with 10% FBS. Cells were grown as monolayers at 37 °C in a humidified CO2 (5%) incubator.

### Drug sensitivity validation

The 8988T, MCF-7, A2780\CP70 and BT474 cells as resistance cells and HCC1806 as sensitive cells were plated into flat-bottomed 96-well plates and cultured in full growth medium, allowing them to attach for 4 hours. Then, the cells were treated with different concentrations of paclitaxel (0–1000 nM), cisplatin (0–1000 µM), and trans-4-methylcyclohexylamine (4MCHA) as SRM inhibitor (4MCHA) (0–1000 µM). The plates were incubated at 37 °C for 72 hours. Cells were treated in triplicate wells for each condition. After the treatment, the cells were washed with PBS, stained with crystal violet (CV) dye for 10 minutes at RT, and washed with distilled water. Plates were left uncovered and air-dried overnight at RT. The next day, 200 µL of the solution containing citrate buffer and 96% ethanol (1w:1w) was added to each well to dissolve the crystal violet stained cells, and incubated for 20 min at RT. The optical density of the extracted dye for each well was measured using an automatic microplate at 570 nm with a reference reading of 0 nm. The cell proliferation percentage was calculated as below:

Cell proliferation = (absorbance of treated cells/ absorbance of untreated cells) × 100

### Drug treatment

For MTT/CTB assays, SRMi (4MCHA) treatment was performed with 5 different doses (18, 53, 110, 251 and 455 uM) for 72 hours. MycMI-6/Niclosamide/Salinomycin treatments were performed with 5 different doses (0.1, 0.3, 1, 3 and 10 uM) for 72 hours. Paclitaxel treatment was performed with 5 different doses (7.21, 9.79, 12, 17 and 29 nM) for 72 hours. Cisplatin treatment was performed with 5 different doses (0.1, 0.3, 1, 3 and 10 uM) for 72 hours. For the combination, each of the above SRMi/ MycMI-6/Niclosamide/Salinomycin and Paclitaxel/cisplatin doses was crossed. The drugs were diluted in DMSO, and therefore DMSO was used as a vehicle control.

For clonogenic assays, SRMi (4MCHA) treatment was performed with 5 different doses (18, 53, 110, 251 and 455 uM) for a week. MycMI-6/Niclosamide/Salinomycin treatments were performed with 5 different doses (0.1, 0.3, 1, 3 and 10 uM) for a week. Paclitaxel treatment was performed with 3 different doses (7, 12 and 17 nM) for a week. Cisplatin treatment was performed with 3 different doses (1, 3 and 10 uM) for a week. For combination, each of the above SRMi/MycMI-6/Niclosamide/Salinomycin and Paclitaxel/cisplatin doses were crossed. The drugs were diluted in DMSO, and therefore DMSO was used as a vehicle control.

For organoids, SRMi was treated in 6 doses (1 mM to 5 mM). Paclitaxel was treated in 5 doses (3 nM to 243 nM). For combination, each of the above SRMi and Paclitaxel doses were crossed. For organoid growth culture, 8 uL BME gel domes were used, 1000 cells/dome were seeded and grown for 7 days before treatment. MTT assay was performed 72 hours after treatment to determine organoid viability. Day 0 corresponds to the day of treatment. Brightfield images were taken on Day 0 and Day 3 before performing the MTT assay.

### Cell titer blue (CTB) cell viability assay

CTB assay was done based on CellTiter-Blue Cell Viability Assay kit (Promega: Cat. No. G8080) according to the manufacturer’s protocol. Briefly, 2500 cells/well were seeded in 96 well plates overnight. After 24 hours, cells were treated with the corresponding concentration of the drugs and incubated for 72 h in the incubator at 37 C. Then, CTB reagent was added and let to incubate for 1–2 hours. Reading was performed using plate reader for CTB setting at 560 and 590 nm.

### Clonogenic growth assay

For clonogenic assay, cell lines were plated at a density of 10,000 cells/well in a T12 dish. Next day, drug treatments were performed. After 1 week, cells were washed once with PBS, and stained with 0.5% Crys[1]tal Violet stain for 10–15 minutes. The stain was removed and washed twice with water. The plates were left to dry overnight. The stain was dissolved in 10% acetic acid and absorbance was read at 595 nm.

### MTT assay

MTT assay was done based on the cell proliferation kit I (Roche: Cat. No. 11 465 007 001) according to the manufacturer’s protocol. Briefly, 2500 cells/well were seeded in 96 well plates overnight. After 24 hours, they were treated with the corresponding concentration of the drugs and incubated for 72 h in the incubator at 37 C. Then, MTT labeling reagent was added and let to incubate for 4 hours. Later, MTT solubilization buffer was added and incubated overnight. Next morning, reading was performed using plate reader for MTT setting at 550 and 600 nm.

### IF staining for FFPE tissues

*Human* colorectal cancer (CRC) and breast cancer non-metastatic/metastatic FFPE tissue sections were purchased from the company Biochain. Tissue sections were deparaffinized in xylene for 10 minutes, followed by immersion in 99% ethanol for 5 minutes, 96% ethanol for 3 minutes, and rinsed in running water for 5 minutes.

For antigen retrieval, the sections were boiled in TEG buffer (10 mM Tris, 0.5 mM EGTA, pH of 9.0.) for 15 minutes, then cooled for 15 minutes before being rinsed with TBS. After drying gently with the tissue paper, a circle was drawn around the FFPE section with a PAP-pen. Blocking of non-specific binding was performed in 1X blocking buffer (For 1 ml: 50 μl horse serum, 250 μl 10% BSA, 700 μl 1X PBS with 0.1% Triton X-100 in PBS) for 2 hours at RT. It was washed with PBS for 5 min. Primary antibodies were diluted in 0.5X blocking buffer (For 2 ml: 1 ml 1x blocking buffer and 1 ml 1X PBS with 0.1% Triton −100). The antibodies used and dilutions applied were as follows: cMYC (9E10), Santacruz, #sc-40, Ms, 1:100; SRM, Proteintech, #19858–1-AP, Rb, 1:500. Primary antibody incubation was performed at 4 °C in a humidified chamber for overnight. Then, the slides were washed three times with PBS during 3 min for each wash. Secondary antibodies were diluted in 0.5X blocking buffer (AF 488 donkey anti-*mouse* IgG (H+L), Thermofisher #A21202, 1:500; AF568 Donkey anti-Rabbit IgG (H+L), Thermofisher #A10042, 1:500) and incubated in a humidified chamber at RT to protect light for 30 min. Next, it was followed with 2 times PBS wash during 3 min each and 2 times ddH2O wash during 5 min each. Mounting was done in 1–2 drops of mounting medium with DAPI (Abcam #ab104139). The slides were then let to dry for 30–60 minutes in the dark and kept in refrigerator. The imaging was performed using confocal microscope (Nikon Confocal A1). Images were quantified using FIJI. In total, 20 independent regions of interest were randomly selected from 5 independent images. Mean intensity for each region of interest were measured, and the data was plotted as a scatter violin plot illustrating the distribution for *cMYC* and *SRM* signal intensity using Graphpad prism.

### Quantitative RT-PCR for SRM upon MYC inhibition

Total RNA of cultured cells was prepared using TRIzol reagent (Invitrogen) and reverse–transcribed with a First Strand cDNA Synthesis kit (Thermofisher). The transcripts were quantified by qPCR using Power-up SYBR Green Master Mix (Applied Biosystems) on a Lightcycler 480 II (Roche). The sequences of the primers used for qPCR are as follows.

SRM_qpcr_F_932 (VT_SDU_932): AAGATGGTGTCCTCTGCTGC

SRM_qpcr_R_933 (VT_SDU_933): TAGGTGGGGATGGTGCAGTA

### Immunofluorescence of tumor organoids with Ki67, PDL-1 and vimentin antibodies

Organoids were cultured in 24 well plates in 70% BME Gel and treated with 9 nM Paclitaxel and 1 mM SRMi for 72 hours. Subsequently, organoids were fixed in 10% formalin for 12 hours, washed with 1× PBS for three times, and treated with 0.2% triton X-100 in 1× PBS for 5 min. The triton x-100 was removed and incubated with blocking reagent (5% goat serum, 1% bovine serum albumin) in 1× PBS for 1 hr. The blocking reagent was removed, and organoids were incubated with Ki67, PDL-1 and Vimentin primary antibody (BioLegend, USA) in a 1:100 dilution) in a cold room overnight. The next day, primary antibodies solution was removed and washed with 1X twin-buffered saline with tween (TBST) for 5 min each for three times. Then organoids were incubated with fluorescently labeled secondary antibody Alexa fluor 488 (ThermoFisher Scientific; Waltham, MA) in a 1:400 dilution. After washing with 1X TBST, organoids were incubated with Hoechst dye 1 μg/ml for 15 min and washed with 1× PBS three times. Finally, 1× PBS was added to the labeled organoids, and fluorescence images were acquired with green, red and 4′,6-diamidino-2-phenylindole (DAPI) acquisition fields.

### In vivo experiment

All animal experiments were performed at the animal facility at University of Southern Denmark and approved by the Animal Experiments Inspectorate of the Ministry of Food, Agriculture and Fisheries of Denmark (2021–15-0201–00843). NOG CIEA *mice* (Taconic) aged between 7 to 9 weeks were acclimatized for one week before initiation of experiments and were housed with ad libitum access to food and water under specific pathogen-free conditions. Tumors were established by subcutaneous injection of 3 × 10^6^ PATU8988T cancer cells in the flank in ECM gel from Engelbreth-Holm-Schwarm murine sarcoma (Sigma-Aldrich). Four days following tumor cell injection *mice* were randomized to receive either vehicle, cisplatin (Weekly with i.p. injection, 5 mg/kg), 4-MCHA (5 days a week by oral gavage, 5 mg/kg) or the combination of cisplatin and 4-MCHA, a dose recently shown to be well tolerated in vivo with stable body weight and minimal toxicity in xenograft models, while achieving effective modulation of polyamine metabolism [35].

### Statistical analysis

All statistical analyses were performed using R (v4.2+), Seurat (v4), and GraphPad Prism (v9), unless otherwise specified. Data are presented as mean ± standard error of the mean (SEM) unless indicated otherwise. All statistical tests were two-sided, and *p* < 0.05 was considered statistically significant. Multiple testing correction was performed using the Benjamini–Hochberg method where applicable.

For single-cell RNA-seq analyses, differential gene expression between chemoresistant and chemosensitive cells was assessed using the Wilcoxon rank-sum test implemented in Seurat, with false discovery rate (FDR) correction. Gene signature and pathway activity scores were compared across resistance states (Low, Medium, High) using Kruskal–Wallis tests followed by pairwise Wilcoxon tests. Associations between pseudotime and gene expression or signature scores were evaluated using Spearman correlation and locally weighted regression (loess) smoothing.

Pathway enrichment analysis (MSigDB hallmark gene sets) was performed using hypergeometric testing, with FDR-adjusted *p*-values used to determine significance. Transcription factor activity and network centrality measures derived from CellOracle were compared using non-parametric tests.

For bulk RNA-seq validation datasets (CTR-DB) and TNMplot analyses, expression differences between groups were evaluated using Wilcoxon rank-sum or Kruskal–Wallis tests, as appropriate. Spatial transcriptomics analyses were conducted using Seurat, and spatial differences in gene expression and module scores across tissue compartments were assessed using non-parametric statistical tests. Correlation analyses were performed using Spearman rank correlation.

ChIP-seq peak significance was determined using a Poisson-based background model with *p*-values adjusted to q-values using the Benjamini–Hochberg method. Peaks were filtered based on predefined FDR thresholds (e.g., q < 1e − 5 or q < 1e−10), and enrichment was quantified using fold enrichment and −log10(q-value) scores.

Survival analyses were performed using the KMplot platform. Kaplan–Meier curves were generated, and statistical significance was assessed using the log-rank test, with hazard ratios (HRs) and 95% confidence intervals reported.

For in vitro assays (MTT, CellTiter-Blue, clonogenic assays), comparisons between two groups were performed using unpaired Student’s t-tests, while multiple group comparisons were analyzed using one-way ANOVA followed by appropriate post hoc tests. When normality assumptions were not met, non-parametric tests were applied. Drug combination effects were evaluated using ZIP and HSA synergy models, and synergy scores were calculated accordingly.

Organoid viability assays were analyzed using two-way ANOVA with multiple comparison correction, and dose–response curves were fitted using nonlinear regression to estimate IC50 values. Immunofluorescence quantification was performed using FIJI, and statistical comparisons between groups were conducted using unpaired t-tests or Mann–Whitney tests.

For in vivo experiments, tumor growth curves were analyzed using two-way repeated measures ANOVA with multiple comparison correction, and endpoint tumor weights were compared using one-way ANOVA. Body weight changes were monitored and analyzed to assess treatment-related toxicity.

## Results

### A distinct cellular and molecular landscape defines chemoresistant status across cancers

**Fig. 1:**
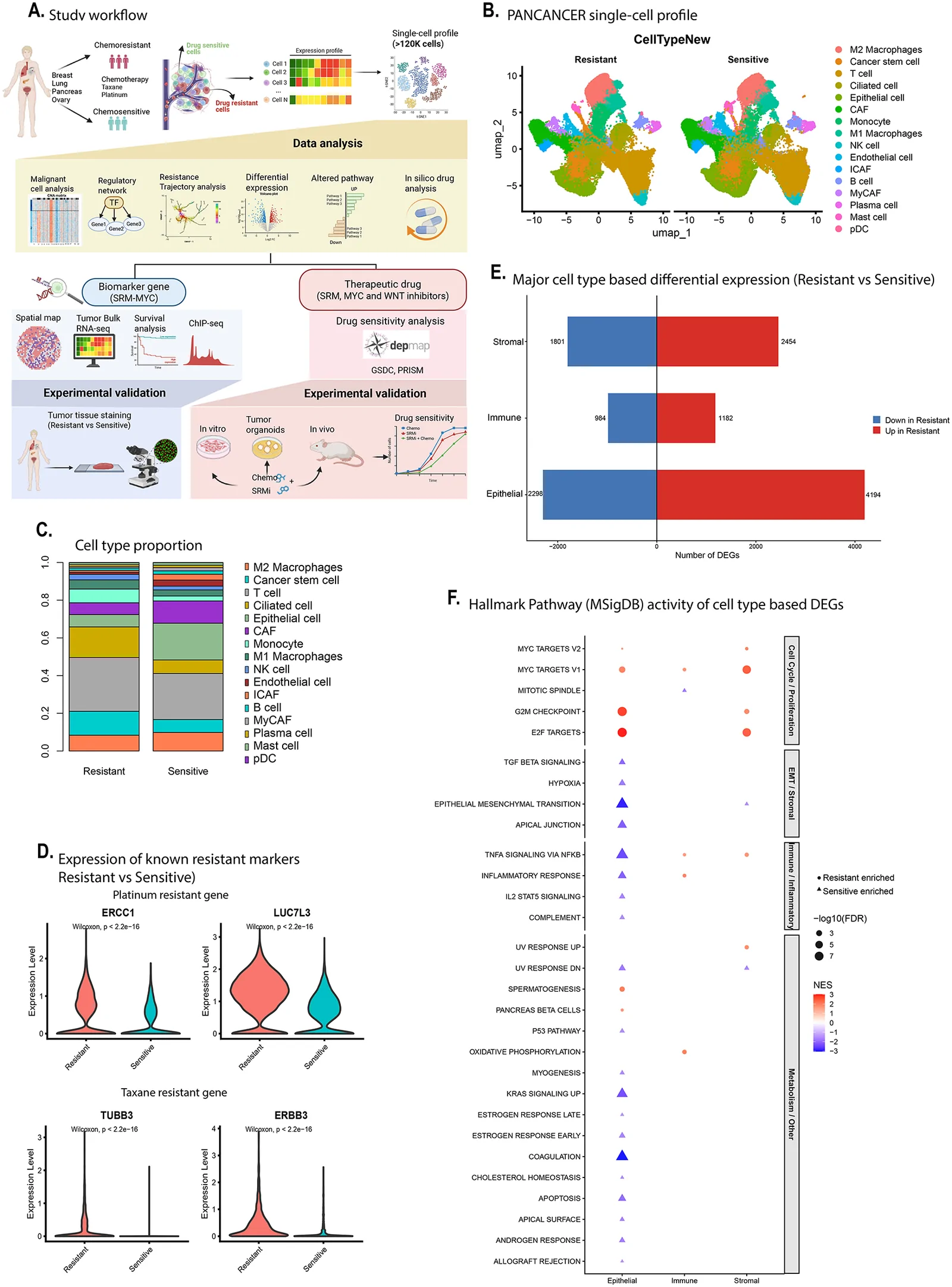
Integrative single-cell pan-cancer analysis identifies conserved chemoresistant states. **A**. Schematic of the study design. Integration of single-cell transcriptomics, spatial transcriptomics, ChIP-seq, and pharmacological screening to dissect mechanisms of chemoresistance across breast, pancreatic, ovarian, and lung cancers. **B**. UMAP projection showing clustering of resistant and sensitive tumor cells across multiple cancer types. Cell types are annotated by color, revealing tumor and stromal heterogeneity. **C**. Bar plot showing the proportion of each cell type in resistant versus sensitive states. **D**. Violin plots showing expression of known resistance genes (*ERCC1, LUC7L3, TUBB3, ERBB3*) in resistant cells across platinum and taxane contexts. Gene expression differences between resistant and sensitive groups were assessed using the two-sided Wilcoxon rank-sum test. **E**. Compartment-specific transcriptional and pathway alterations associated with chemoresistance. Differentially expressed genes (DEGs) between resistant and sensitive cells within epithelial, immune, and stromal compartments. Horizontal diverging bar plots show the number of genes significantly upregulated in resistant cells (red) and downregulated in resistant cells (blue). Numbers indicate the total count of significant DEGs in each compartment. **F**. MSigDB hallmark pathway enrichment analysis comparing resistant and sensitive cells across epithelial, immune, and stromal compartments. Bubble size represents pathway significance (−log10 FDR), color indicates normalized enrichment score (NES), and shape denotes enrichment direction (circles, resistant-enriched; triangles, sensitive-enriched). Hallmark pathways are grouped into cell Cycle/Proliferation, EMT/Stromal, immune/Inflammatory, and Metabolism/Other functional categories

To unravel the tumor heterogeneity in aggressive cancers, we analyzed single-cell transcriptomes from 29 tumors, both chemoresistant and chemosensitive, across four major cancer types: Breast [7, 8], Lung [9, 10], Ovary [11], and Pancreas [12, 13] (Fig. 1A). Here, all patients were treated with platinum, Folfirinox and or taxane-based chemotherapies either alone or in combination (Additional file 2: Table S1). The analytical framework employed encompassed clonal analysis, regulatory network reconstruction, cell–cell communication analysis, pathway dysregulation mapping, and drug repurposing pipelines, followed by experimental validation in vitro and in Patient-Derived models.

After rigorous quality control, we analyzed 126,724 cells, divided between chemoresistant (60,171 cells) and chemosensitive (66,553 cells) tumors. To account for data variability across independent studies, we used canonical correlation analysis (CCA) to eliminate batch effects and successfully integrated the datasets. This integration revealed 16 distinct cell types with notable differences in their distribution between resistant and sensitive tumors (Fig. 1B).

Our analysis revealed that all major immune, stromal, and epithelial compartments were preserved across both phenotypes, allowing direct comparisons within matched cell types. Notably, while global clustering appeared similar, finer transcriptional distinctions emerged upon deeper interrogation. Quantitative assessment of cell-type proportions revealed enrichment of cancer stem cells in chemoresistant samples compared to sensitive ones (Fig. 1C). Interestingly, epithelial populations remained stable in resistant tumors but dropped in sensitive tumors upon treatment, potentially reflecting the regeneration of normal epithelial cells. Consistent with known biology, resistant tumors exhibited significantly higher expression of well-established chemoresistance genes. Platinum-resistance markers *ERCC1* and *LUC7L3*, and taxane-resistance markers *TUBB3* and *ERBB3*, were strongly upregulated in the resistant state (Fig. 1D). These findings further confirmed that these cells are resistant to cisplatin and taxane chemotherapies.

To identify compartment-specific programs associated with chemoresistance, we grouped cells into epithelial, immune, and stromal compartments and performed differential expression analysis between resistant and sensitive cells. The epithelial compartment exhibited the greatest transcriptional changes, with 4,194 genes upregulated and 2,298 genes downregulated in resistant cells. Stromal cells also showed substantial remodeling (2,454 upregulated and 1,801 downregulated genes), whereas immune cells displayed comparatively fewer alterations (1,182 upregulated and 984 downregulated genes) (Fig. 1E).

Hallmark pathway enrichment analysis using MSigDB [36] revealed strong activation of cell-cycle and proliferation-related programs, including E2F Targets, G2M Checkpoint, MYC Targets, and Mitotic Spindle, within resistant epithelial and stromal compartments. In contrast, pathways associated with EMT, hypoxia, and inflammatory responses were predominantly enriched in sensitive cells (Fig. 1F). Together, these results demonstrate distinct compartment-specific pathway alterations associated with the resistant state.

### Trajectory of chemoresistance reveals emergence of stemness and proliferative potential in resistant cells

**Fig. 2:**
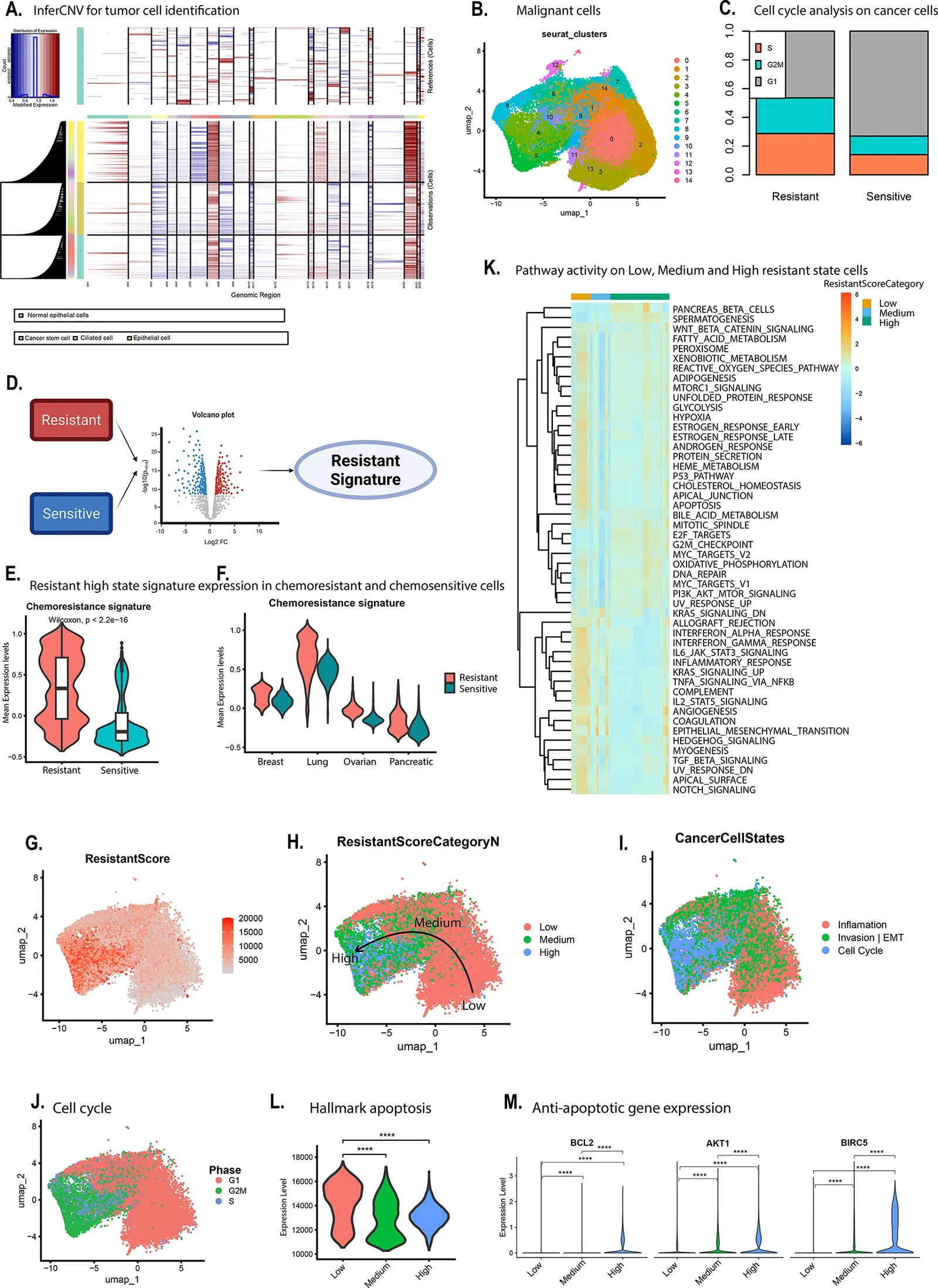
Identification and characterization of chemoresistant cell states. **A.** The inferCNV analysis of the integrated PANCANCER dataset. The upper heatmap plot shows the copy number alteration profile in normal epithelial cells. The lower heatmap plot shows CNV profile in the integrated dataset. We have used a total of 240 normal epithelial cells to estimate somatic copy number alteration in PANCANCER epithelial, cancer stem cells, and ciliated cells. Regions of chromosomal amplification manifest as blocks of red, while chromosomal deletions manifest as blue blocks, providing a visual representation of the copy number changes. **B.** UMAP projection of malignant cells with resistant and sensitive cluster labels. **C.** Stacked bar plot of cell cycle phases in resistant versus sensitive tumor cells. Resistant cells are enriched in S and G2M phases. Cell-cycle states were inferred using the CellCycleScoring() function implemented in Seurat (v5). **D.** Differential gene expression analysis between resistant and sensitive malignant cells. Volcano plot highlights genes associated with the chemoresistant transcriptional program. **E.** Violin plot showing expression of resistant signature in resistant versus sensitive cells. Gene expression differences between resistant and sensitive groups were assessed using the two-sided Wilcoxon rank-sum test. **F.** Expression of the chemoresistance signature across breast, lung, ovarian, and pancreatic cancer cells. **G.** UMAP visualization of continuous chemoresistance scores across malignant cells. **H.** UMAP with cells categorized into low, medium, and high resistance states, overlaid with inferred pseudotime trajectory. **I.** Functional cancer cell-state annotation using CancerSEA signatures. Cells were classified according to dominant functional programs, including cell cycle, inflammation, and invasion/EMT. **J.** UMAP showing the distribution of cell-cycle phases across malignant cells. Cell-cycle states (G1, S, and G2/M) were assigned using the CellCycleScoring() function implemented in Seurat, which utilizes curated S-phase and G2/M-phase gene signatures originally described by Tirosh et al. 2016 [19]. **K.** Heatmap of hallmark pathway activity across low-, medium-, and high-resistance states. Resistant cells are characterized by increased MYC signaling, E2F targets, DNA repair, G2/M checkpoint, and PI3K–AKT–mTOR pathway activity. **L.** Violin plots showing decreasing apoptosis signature from low-, medium-, and high-resistance states. Resistant state differences were assessed using two-sided Wilcoxon rank-sum tests, with *p* values shown on the plot. **M.** Expression of anti-apoptotic genes (*BCL2, AKT1*, and *BIRC5*) across low-, medium-, and high-resistance malignant cells. Violin plots show single-cell expression distributions. Statistical comparisons between groups were performed using the Wilcoxon rank-sum test, and significance levels are indicated on the plots (*p* < 0.05, *p* < 0.01, *p* < 0.001, *p* < 0.0001)

Given the diverse cellular composition of the tumor microenvironment, our next objective was to distinguish malignant and non-malignant epithelial cells. To identify malignant cells, we leveraged the inferCNV package [15], which detects copy number variations (CNVs) such as chromosomal gains or deletions, by comparing them to a reference set of normal cells. Our CNV analysis revealed that the majority of epithelial, ciliated, and cancer stem cells exhibited significantly elevated CNV profiles (Fig. 2A, bottom heatmap) when compared to normal reference epithelial cells from a previous study [37] (Fig. 2A, upper heatmap). This analysis identified a total of 42,765 malignant cells based on their heightened CNV levels relative to the reference epithelial cells within the integrated dataset. We then extracted these malignant cells from both chemoresistant and chemosensitive tumors and re-clustered them for further downstream analysis (Fig. 2B).

Additionally, we characterized the cancer cells by their cell cycle states, distinguishing between proliferative (cycling) and non-proliferative (non-cycling) phases. Strikingly, most cancer cells in chemoresistant patients were in the G2M/S phase (Fig. 2C), indicative of active cycling or proliferation. In contrast, cancer cells in chemosensitive tumors predominantly resided in the G1 phase, representing a non-cycling, quiescent state (Fig. 2C). In summary, cell cycle phase distribution showed a significant enrichment of resistant cells in S and G2/M phases, suggesting they are proliferating, not dormant. Next, we sought to identify the core PANCANCER resistant signature and performed differential gene expression analysis between resistant and sensitive cells (Fig. 2D). The differential analysis identified total of 256 and 249 genes that are upregulated and downregulated in chemoresistant cells (Additional file 2: Table S5–S6). To remove cancer-dependent bias, we selected genes that are consistently upregulated in chemoresistant cells of all four cancers and identified a total of 100 genes as the PANCANCER core chemoresistant gene signature (Fig. 2E–F, Additional file 2: Table S4). Next, we extracted cancer cells of the resistant type from the integrated dataset and scored each cell using the core chemoresistant signature and, interestingly, found a trajectory-like pattern (Fig. 2G). This signature stratified cells into Low, Medium, and High resistance states, forming a gradient of resistance progression (Fig. 2H). This stratification revealed a continuous resistance axis, rather than a binary distinction, suggesting that tumor cells gradually acquire or amplify resistance properties rather than switching states abruptly.

We next projected resistance scores onto annotated cancer cell states (Fig. 2I) and surprisingly, the Cell Cycle cluster exhibited high resistance scores in the chemoresistance category map, in contrast to the common assumption that cycling cells are more sensitive to cytotoxic agents. In contrast, the EMT/Invasive and Inflammatory clusters were predominantly populated by cells with low to medium resistance scores. Notably, cell cycle phase scoring indicated that these resistant cells were predominantly in S and G2/M phases, highlighting an actively cycling, mitotically engaged subpopulation rather than a dormant or quiescent one (Fig. 2J). These findings challenge prevailing paradigms which link chemoresistance primarily to dormant or plastic cell states, such as EMT [38].

Pathway activity profiling across resistance categories (Fig. 2K) identified strong enrichment of E2F targets, MYC targets, G2M checkpoint, DNA repair, and oxidative phosphorylation in high-resistance states. These programs collectively support a phenotype of mitotic competence, metabolic fitness, and damage repair, enabling cells to withstand chemotherapy-induced stress. Importantly, the enrichment of PI3K/AKT/mTOR signaling, also observed in other datasets, suggests that these resistant cells activate parallel survival pathways that augment cell cycle re-entry and DNA repair, further insulating them from drug effects.

Although resistant cells activate damage-response pathways, they maintain a suppressed apoptotic profile, as indicated by low HALLMARK_APOPTOSIS scores in high-resistance cells (Fig. 2L). At the gene level (Fig. 2M), expression of key anti-apoptotic factors, *BCL2*, *AKT1*, and *BIRC5* (Survivin), was strongly elevated in high-resistance cells. This profile suggests a decoupling of DNA damage sensing from apoptosis, enabling resistant cells to proliferate despite genotoxic injury, a hallmark of cancer stem-like behavior.

### Therapy‑driven selection and evolution of a transcriptionally and genomically distinct chemoresistant state

**Fig. 3:**
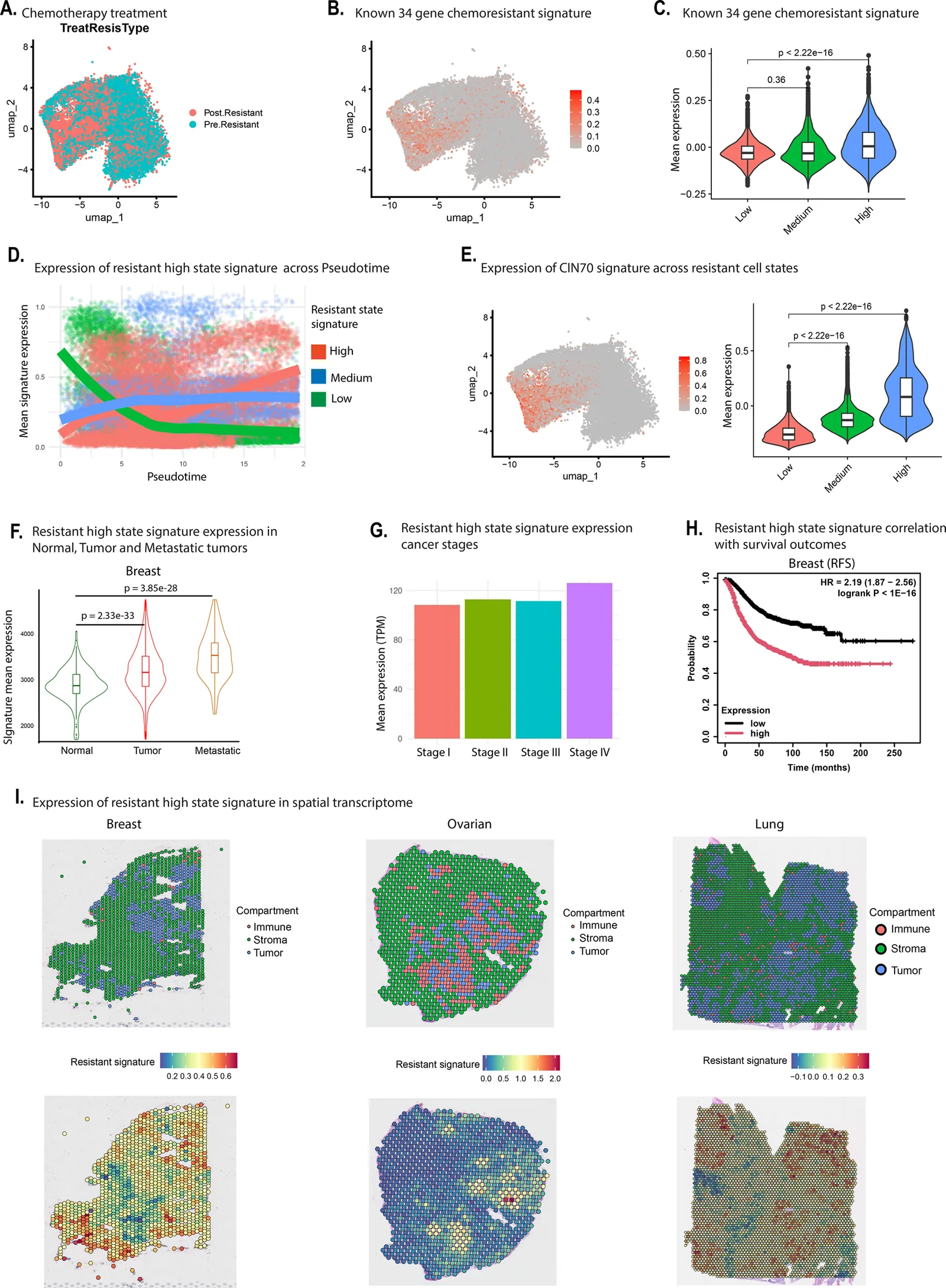
High-resistant cells display proliferative features, chromosomal instability, and spatial localization. **A.** UMAP projection of malignant cells colored by treatment status (pre-treatment versus post-treatment). **B.** UMAP showing expression of previously reported 34-gene chemoresistance signature across resistance score groups (Supplementary Table S7). **C.** Distribution of the 34-gene chemoresistance signature across low-, medium-, and high-resistance states. Statistical comparisons were performed using two-sided Wilcoxon rank-sum tests, with *p* values indicated on the plot. **D.** Pseudotime trajectory with overlay of chemoresistance signature expression. Resistant cells occupy terminal high-resistance states. **E.** CIN70 chromosomal instability signature across resistance states. Left: UMAP showing CIN70 signature expression. Right: violin plots comparing CIN70 scores across low-, medium-, and high-resistance cells. Statistical comparisons were performed using two-sided Wilcoxon rank-sum tests. **F.** Violin plots of resistant signature in normal, tumor, and metastatic tissues. The group differences across normal, tumor, and metastatic samples were derived from TNMPlot and assessed using the Kruskal–Wallis test followed by Dunn’s post hoc multiple comparisons test, with adjusted *p* values reported. **G.** Boxplots of resistant signature by clinical stage (Stage I–IV). **H.** Kaplan–Meier survival curves stratified by signature-high vs low in breast cancer cohorts available at KMPlotter. Survival differences were evaluated using the log-rank (Mantel–Cox) test, with hazard ratios estimated using Cox proportional hazards models. **I.** Spatial transcriptomic mapping of the chemoresistance signature in breast, ovarian, and lung cancer tissues. Spots are annotated as tumor, stromal, or immune compartments, and colored according to chemoresistance signature expression

Following the characterization of chemoresistance as a proliferative, transcriptionally distinct state (Fig. 2), we next examined how this state is shaped by therapy. UMAP overlays of pre- and post-treatment tumor cells (Fig. 3A) showed that post-chemotherapy samples are highly enriched in the High-resistance subpopulations, whereas pre-treatment cells were more broadly distributed across the Low and Medium resistance zones. This suggests that chemotherapy acts as a selective pressure, amplifying a pre-existing resistant subpopulation with favorable survival traits. This transition was further validated by the projection of a known 34-gene chemoresistance signature (Fig. 3B, Additional file 2: Table S7), which was highly enriched in resistant zones. Furthermore, this external signature showed graded enrichment across Low, Medium, and High resistance states (Fig. 3C, *p* < 2.2e − 16), confirming the biological and clinical relevance of the defined resistance program.

To determine whether resistance arises abruptly or evolves progressively, we constructed a pseudotime trajectory across tumor cells (Fig. 3D). Notably, the expression of the resistance signature increased continuously along pseudotime, with High-resistance cells appearing at later stages of the trajectory. This indicates that chemoresistance is a dynamically acquired phenotype, likely reinforced over time by therapy-induced selective pressures, transcriptional plasticity, or both.

Given that cancer cells often exhibit chromosomal instability (CIN) and enhanced resistance to therapies [39, 40], We next investigated whether genomic features underlie or support the resistant state. We employed the CIN70 signature, a marker of chromosomal instability correlated with increased aneuploidy across various cancer types [41]. Mapping CIN70 scores, a surrogate for chromosomal instability, revealed stepwise elevation across resistance strata, peaking in High-resistance cells (Fig. 3E). This suggests that resistant cells are not only transcriptionally distinct but also genomically unstable, potentially leveraging aneuploidy tolerance and DNA repair activation to support survival under genotoxic stress.

We assessed the expression of the resistance signature across tissue types using transcriptomic data from all four cancer types. Violin plot analysis showed significantly higher signature expression in primary tumors and metastatic lesions compared to adjacent normal tissue (Fig. 3F, Additional file 1: Fig. S1A), confirming that the resistance state is intrinsic to malignant cells and retained during metastatic progression.

Contrary to the notion that resistance arises only in late-stage or relapsed tumors, analysis of breast cancer clinical cohorts revealed stable expression of the resistance signature across all tumor stages (Fig. 3G). This suggests that the chemoresistant state can emerge early in tumorigenesis and persist throughout disease progression, raising the possibility that even early-stage tumors harbor subclones capable of therapy escape.

To evaluate its prognostic relevance, we stratified patients based on resistance signature expression and performed Kaplan–Meier survival analysis. Patients with high signature expression experienced significantly worse relapse-free survival (Fig. 3H; HR = 2.19, *p* < 1e − 16) across these cancers (Additional file 1: Fig. S1B), strongly linking this transcriptional program to treatment failure and disease recurrence. These results position the signature as both a biomarker for risk stratification and a target for therapeutic intervention.

To validate the spatial localization of the chemoresistance program, we analyzed publicly available spatial transcriptomic datasets of aggressive patients from breast [21], pancreatic [22], ovarian [23], and lung cancers (10x Genomics Visium dataset). Finally, spatial transcriptomic profiling across multiple tumor types revealed that expression of the resistance signature was strongly localized to tumor epithelial regions (Fig. 3I). In contrast, immune and stromal compartments exhibited low to negligible expression of this signature. This spatial localization supports the hypothesis that resistance arises not from the tumor microenvironment or immune evasion, but from an intrinsically resistant epithelial subpopulation, likely shaped by clonal selection during treatment. These findings, in conjunction with those from Fig. 2, illustrate a coherent, multifaceted model of chemoresistance as a spatiotemporally defined, genomically unstable, proliferative tumor state, which emerges under treatment, persists across time and space, and correlates with adverse clinical outcomes.

### Transcription factor activity identifies MYC as a key pan-cancer driver of the chemoresistant cell state

**Fig. 4:**
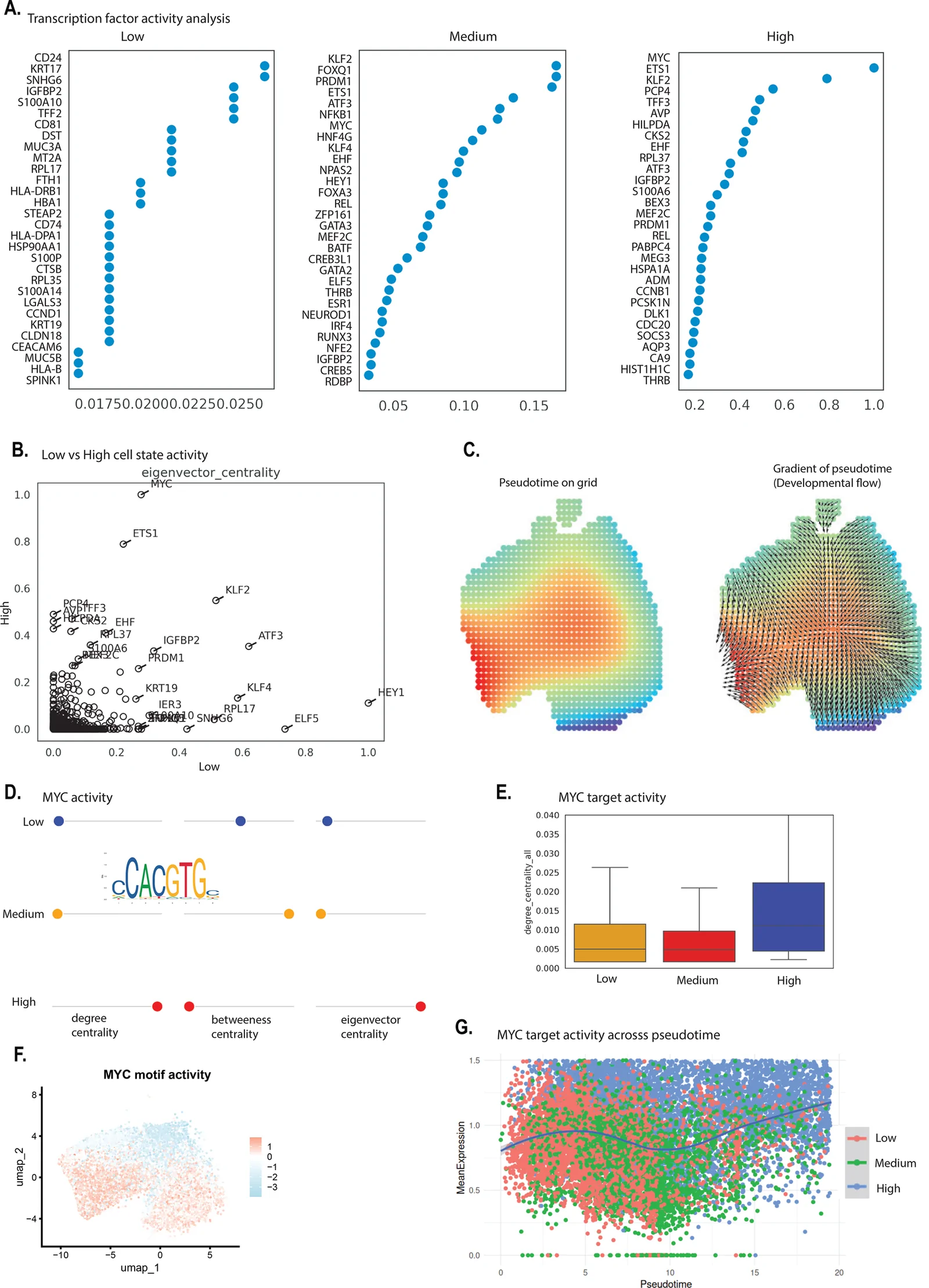
MYC activity defines resistance high cell state trajectories. **A.** CellOracle gene regulatory network analysis showing the top transcriptional regulators across low-, medium-, and high-resistance states. Regulators are ranked according to inferred network activity scores. **B.** Network centrality analysis comparing low- and high-resistance states. Each point represents a transcription factor positioned according to its eigenvector centrality within the inferred regulatory network. **C.** CellOracle-based pseudotime and developmental flow analysis. Left: cells projected onto a pseudotime grid and colored according to inferred pseudotime values. Right: developmental flow field inferred from CellOracle gene regulatory network analysis. Arrows indicate the predicted direction and magnitude of transcriptional state transitions. **D.** CellOracle-based regulatory network centrality analysis of MYC across low-, medium-, and high-resistance cell populations, showing degree, betweenness, and eigenvector centrality scores. The MYC motif logo shown centrally is the canonical MYC binding motif from the JASPAR database and is included for visualization only. **E.** Boxplot plots showing expression of MYC direct target genes across resistance states. **F.** UMAP highlighting MYC motif activity across malignant cells. **G.** MYC target activity scores were calculated across all cells along the pseudotime trajectory. The plotted values represent the mean MYC target score across cells within the low-, medium-, and high-resistance groups

To understand the transcriptional regulators governing the emergence of resistance, we performed network centrality analyses across tumor cell regulatory networks stratified by resistance states using the CellOracle tool [27]. Transcription factor (TF) activity inference across resistance states (Fig. 4A) revealed a progressive activation of MYC along the Low → Medium → High resistance continuum. Alongside MYC, several additional transcriptional regulators, including ETS1, KLF2, PCP4, TFF3, AVP, HILPDA, CKS2, EHF, and ATF3, showed elevated activity in the High-resistance group (Fig. 4A). However, MYC consistently exhibited the strongest activity and network connectivity among resistance-associated regulators. Scatter plots of eigenvector centrality further highlighted MYC as one of the highest-ranking nodes within the transcriptional network, together with ETS1, KLF2, ATF3, and EHF (Fig. 4B). These findings suggest that the resistant phenotype is governed by a coordinated transcriptional hierarchy, with MYC serving as a central regulator that integrates proliferative, metabolic, and survival-associated programs during resistance acquisition. This reinforces earlier pathway-level findings (Fig. 2K) and validates that the resistance state is not only defined by MYC target gene activation but is coordinated by a hierarchical regulatory network with MYC at its apex. These nodes are likely to propagate regulatory influence efficiently across the transcriptional network, placing MYC in a topologically privileged position to coordinate resistance-associated programs.

To reconstruct the temporal dynamics of resistance acquisition, we applied pseudotime analysis across tumor cells (Fig. 4C). The pseudotime grid and developmental flow plots showed a clear directional trajectory from Low-resistance to High-resistance states, with smooth transitions suggesting transcriptional plasticity rather than abrupt fate switches. This developmental gradient aligns with prior analyses of cell state transitions (Figs. 2–3) and highlights a progressive adaptation to chemotherapeutic stress. Gene regulatory network analysis demonstrated progressive increases in MYC network centrality across the resistance trajectory. MYC exhibited elevated degree, betweenness, and eigenvector centrality scores in High-resistance cells compared with Low- and Medium-resistance populations, indicating that MYC occupies an increasingly influential position within the resistance-associated regulatory network (Fig. 4D). Quantitative motif activity showed a progressive increase from Low to High resistance groups, reinforcing that TF engagement, particularly by MYC, scales with chemoresistant identity (Fig. 4E). UMAP projection of MYC motif activity revealed strong enrichment within the High-resistance cluster (Fig. 4F), mirroring earlier findings on MYC expression and TF activity. This underscores the consistency between MYC’s transcriptional, regulatory, and spatial footprint in the resistant state. Finally, analysis of MYC target activity across pseudotime revealed a progressive increase along the resistance trajectory, consistent with enhanced MYC-regulated transcriptional programs during acquisition of the chemoresistant state (Fig. 4G). This provides temporal support for MYC’s role in driving resistance-state acquisition, as MYC expression precedes and tracks with the emergence of resistant phenotypes.

Together, these data establish that the transition toward chemoresistance is coordinated through a structured regulatory hierarchy dominated by MYC, which serves as both a topological hub and dynamic effector. This observation is also consistent with its established role in promoting cell-cycle progression and cellular proliferation by regulating genes involved in cell-cycle control [42]. The integration of centrality analysis, motif accessibility, pseudotime trajectory, and gene expression confirm that MYC orchestrates the stepwise evolution of resistant tumor cell states.

### SRM is a novel pan-cancer marker for chemoresistance

**Fig. 5:**
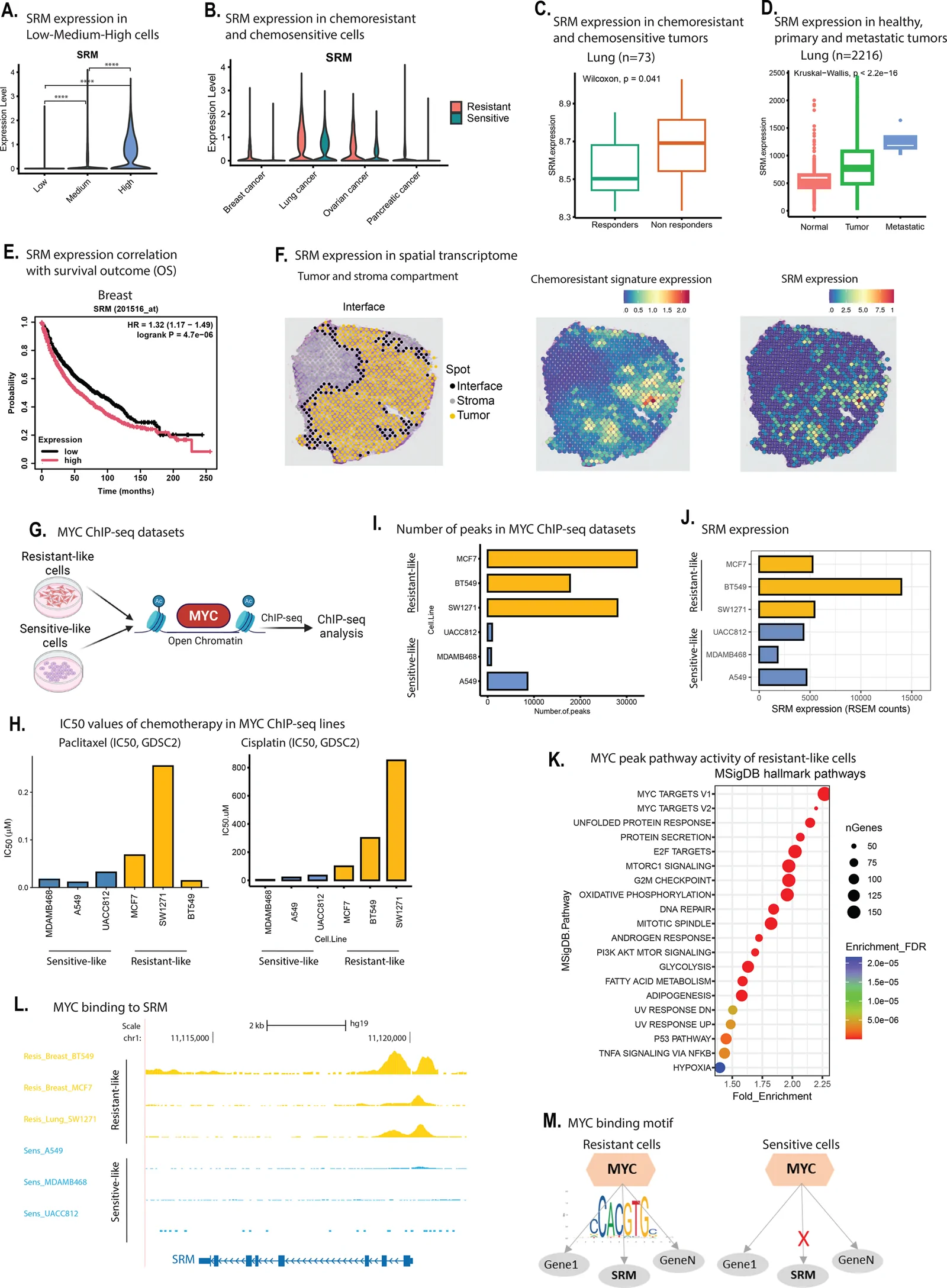
SRM is a MYC-regulated marker of chemoresistant cell states. **A.** SRM expression across low-, medium-, and high-resistance malignant cells. **B.** SRM expression in resistant and sensitive tumor cells across breast, lung, ovarian, and pancreatic cancers. **C.** Boxplot of *SRM* in cisplatin-resistant vs sensitive tumors. The box-and-whisker plots, the horizontal lines mark the median, the box limits indicate the 25th and 75th percentiles, and the whiskers extend to 1.5× the interquartile range from the 25th and 75th percentiles. The statistical testing of expression levels of signature genes between the groups was performed using two-tailed unpaired Wilcoxon test. **D.** *SRM* expression across normal, primary, and metastatic tumors in lung datasets. The TCGA pan-cancer RNA-seq dataset was retrieved from TNMplot web-based application. Group differences across normal, tumor, and metastatic samples were assessed using a Kruskal–Wallis test, with *p* values shown on the plot. **E.** Kaplan–Meier survival analysis for breast cancer patients with high SRM. Survival differences were evaluated using the log-rank (Mantel–Cox) test, with hazard ratios estimated using Cox proportional hazards models. **F.** Spatial transcriptomic analysis of SRM expression. Left: tumor, stromal, and interface compartments. Middle: chemoresistance signature expression. Right: SRM expression in the corresponding tissue section. **G.** Experimental design of MYC ChIP-seq in resistant vs sensitive cells. The ChIP-seq datasets were derived from ChIP-Atlas database database. **H.** Barplots of paclitaxel/cisplatin IC_50_ values in MYC-high vs MYC-low cells. The drug IC_50_ values of these cell lines were derived from Genomics of drug sensitivity in cancer (GDSC) project. **I.** Number of MYC ChIP-seq peaks identified in resistant-like and sensitive-like cell lines. **J.** SRM expression in resistant-like and sensitive-like cancer cell lines. SRM mRNA expression levels in representative resistant-like (MCF7, BT549, and SW1271) and sensitive-like (A549, MDAMB468, and UACC812) cancer cell lines identified by the machine learning classifier. Expression values were obtained from CCLE RNA-seq data (RSEM counts). **K.** Enrichment of MYC targets in MSigDB hallmark pathways in resistant-like cells. MYC peaks were annotated and nearest genes of these peaks were subjected for MSigDB hallmark pathway enrichment analysis. **L.** Genome browser snapshot showing MYC occupancy at the SRM locus. The browser track shows *MYC* binding to *SRM* gene in resistant-like (yellow) and sensitive-like (blue) cell lines. **M.** Proposed model of MYC-mediated transcriptional regulation of *SRM* in chemoresistant cells

Next, we investigated novel genes specifically enriched in the highly resistant state by comparing chemoresistant and chemosensitive tumors across all cancer types, aiming to identify candidates that may drive chemoresistance. We thoroughly looked at the resistant signature genes obtained from Fig. 2D and subjected these genes to a systematic large-scale analysis viz. *1) multi-cancer, 2) multi-cohort (a large number of patients), 3) cross-platform (bulk approach-based dataset), 4) multi-modal (Spatial transcriptome),* and *5) thoroughly conducted literature search*. Interestingly, this in-depth analysis identified *SRM* (Spermidine Synthase) as a novel pan-cancer marker of chemoresistance, meeting all defined criteria with high confidence. For example, *SRM* showed higher expression in the high-resistance population, with minimal expression in lower-resistance states (Fig. 5A). Furthermore, *SRM* expresses at higher levels in resistant cells of all four cancer types in our single-cell dataset as compared to the sensitive ones (Fig. 5B).

Given the elevated *SRM* activity observed in chemoresistant tumors within our single-cell datasets, we sought to validate these findings in larger patient cohorts. To this end, we analyzed available bulk RNA-seq datasets from chemoresistant and chemosensitive tumors across four cancer types, Breast [43], Lung [44], Ovarian [45], and Pancreatic cancers [46]. Notably, our analysis revealed a significantly higher expression of *SRM* in chemoresistant tumors compared to their chemosensitive counterparts, underscoring the robustness of *SRM* as marker of chemoresistance across independent cohorts (Fig. 5C, Additional file 1: Fig. S1C). Furthermore, *SRM* was significantly overexpressed in primary tumors compared to matched normal tissues across multiple cancer types, with expression levels further elevated in metastatic tumors (Fig. 5D, Additional file 1: Fig. S1D). Strikingly, elevated *SRM* expression was strongly associated with poor patient survival outcomes, further reinforcing its potential as a key prognostic biomarker and therapeutic target in chemoresistant cancers (Fig. 5E, Additional file 1: Fig. S1E).

Spatial transcriptomics enables the identification of distinct cell types and states within tumors and their microenvironment while preserving spatial context. To investigate the spatial expression of SRM within the tumor microenvironment, we leveraged spatial transcriptomic datasets from aggressive tumors across four cancer types, i.e. Breast [21], Lung (10x Genomics Visium dataset), Ovary [23], and Pancreas [22]. Importantly, the breast, pancreatic, and ovarian datasets were generated from clinically aggressive tumors, including treatment-resistant, residual, recurrent, or advanced disease samples, making them particularly relevant for investigating chemoresistance-associated transcriptional programs. For spatial analysis, we first delineated malignant cells within tissue sections using the SpaCET tool, which estimates cellular identities and stratifies tumor and stromal compartments (Fig. 5F, left panel). Next, we assessed which malignant cells exhibited chemoresistant properties by applying a curated platinum-resistance gene signature consisting of 317 genes [47] (Fig. 5F, middle panel). Strikingly, SRM expression was highly enriched within the malignant compartment exhibiting chemoresistant characteristics, a pattern that was consistently observed across all cancer types (Fig. 5F, right panel, Additional file 1: Fig. S2A). These findings establish SRM as a core marker of malignancy and chemoresistance, highlighting its strong clinical potential as a therapeutic target for overcoming chemoresistance across multiple cancer types.

Given our gene regulatory network analysis of the highly resistant state identified MYC as one of the top factors, we further explored whether MYC regulates SRM levels in aggressive tumors. Previous studies have demonstrated that MYC collaborates with polyamine synthesis genes to drive cell-cycle progression, cellular proliferation, and malignant transformation [37, 42, 48–50]. To investigate this, we analyzed MYC ChIP-seq datasets (Fig. 5G) from three cell lines to exhibit poor (resistant-like) and three cell lines with good (sensitive-like) responses to taxol and platinum-based chemotherapy. We confirmed the sensitivity of these cell lines to platinum (Fig. 5H, right barplot) and taxol (Fig. 5H, left barplot) treatments by assessing their IC_50_ values. As anticipated, resistant-like cells, characterized by high MYC activity and elevated SRM levels, exhibited higher IC_50_ values, indicating a reduced sensitivity to chemotherapy.

Further analysis revealed that MYC binds a significantly larger number of loci in resistant cells while this was notably diminished in sensitive cells, indicating a stronger transcriptional role in aggressive cells (Fig. 5I). To further investigate the relationship between MYC activity, SRM expression, and chemoresistance, we selected representative resistant-like and sensitive-like cell lines for downstream mRNA expression analyses. *SRM* expression was generally elevated in resistant-like (MCF7, BT549, and SW1271) compared with sensitive-like cell lines (A549, MDAMB468, and UACC812), although the magnitude of expression varied among individual models (Fig. 5J). Similarly, resistant-like cell lines generally showed reduced paclitaxel sensitivity, but this relationship was not uniform across individual models, with BT549 displaying a comparatively low paclitaxel IC_50_ despite its resistant-like molecular profile (Fig. 5H). This heterogeneity indicates that steady-state *SRM* expression and the MYC–SRM-associated molecular state do not translate into a proportional increase in chemotherapy IC_50_ across all cell lines. Rather, these findings support a context-dependent relationship in which MYC-associated transcriptional activity contributes to the chemoresistant state, while drug sensitivity is additionally influenced by cell-line- and drug-specific factors.

Functional pathway analysis of MYC-bound genes revealed an enrichment of pathways related to cell cycle regulation (MYC, G2M, E2F), DNA repair, mTOR, NFκB, and p53 in resistant cells, but not in sensitive cells (Fig. 5K). These findings are consistent with our earlier observations of heightened cell-cycle activity and enhanced DNA repair mechanisms in resistant cells (Fig. 2I–L). Importantly, analysis of MYC occupancy at the *SRM* locus revealed preferential MYC binding in resistant-like cell lines compared with sensitive-like models (Fig. 5L–M), supporting a direct transcriptional regulation of SRM as one component of the broader MYC-associated resistance program.

### SRM and MYC exhibit elevated expression and colocalize in chemoresistant tumors

**Fig. 6:**
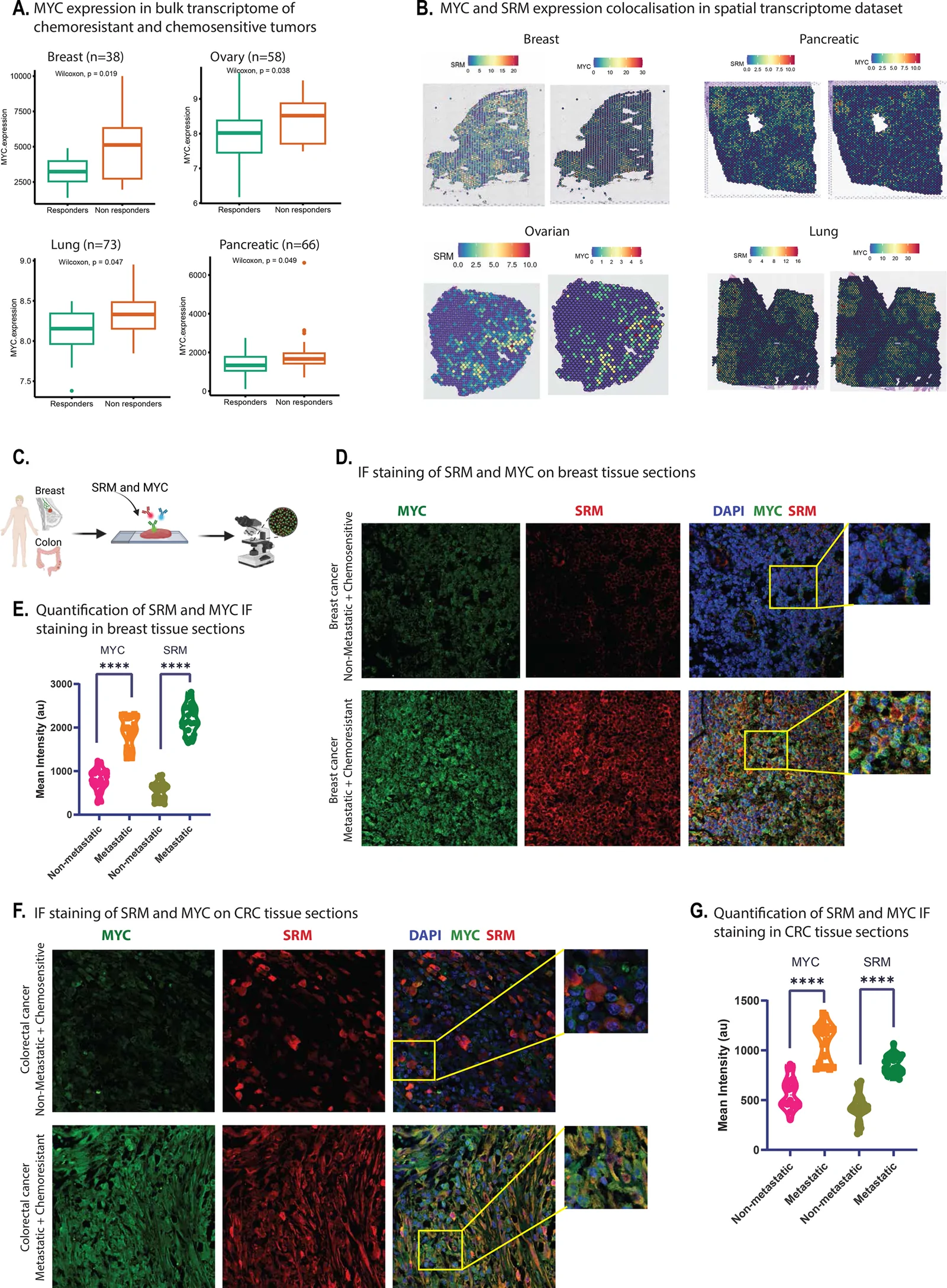
MYC and SRM co-expression defines chemoresistant tumors in spatial and tissue contexts. **A.** Boxplots of MYC and SRM expression in responders vs non-responders across four cancer datasets. The box-and-whisker plots, the horizontal lines mark the median, the box limits indicate the 25th and 75th percentiles, and the whiskers extend to 1.5× the interquartile range from the 25th and 75th percentiles. The statistical testing of expression levels of signature genes between the groups was performed using two-tailed unpaired Wilcoxon test. **B.** Spatial transcriptomic mapping of *MYC* and *SRM* expression across breast, ovarian, pancreatic, and lung cancer tissues. **C.** Experimental workflow for tissue-level validation of *MYC* and *SRM* expression using co-immunofluorescence staining in breast and colorectal cancer (CRC) specimens. **D.** Representative immunofluorescence images of *MYC* and *SRM* expression in chemosensitive/non-metastatic and chemoresistant/metastatic breast cancer tissues. Insets show higher magnification views of the highlighted regions. **E.** Quantification of MYC/SRM immunofluorescence signal intensity in breast cancer tissue sections. **F.** Representative immunofluorescence images of *MYC* and *SRM* expression in chemosensitive/non-metastatic and chemoresistant/metastatic colorectal cancer tissues. Insets show higher magnification views of the highlighted regions. Scale bar, 20 μm. **G.** Quantification of IF staining across chemosensitive + non-metastatic and chemoresistant + metastatic CRC samples. For statistical analysis, student ‘s t test was performed (**** *p* < 0.0001)

We next examined whether MYC levels are elevated and spatially co-localize with SRM within tumor sections from chemoresistant patients. To begin, we first analyzed *MYC* expression in bulk transcriptome datasets from chemoresistant and chemosensitive tumors across four cancer types [43–46]. This analysis revealed a significant upregulation of *MYC* in chemoresistant tumors compared to their chemosensitive counterparts (Fig. 6A). To further investigate the spatial relationship between MYC and SRM, we leveraged spatial transcriptome datasets from chemoresistant patients (shown in Fig. 3I). Strikingly, our spatial analysis demonstrated elevated *MYC* mRNA levels within the tumor compartment, the same regions where SRM is highly expressed, indicating their co-localization in aggressive cancers (Fig. 6B).

To validate these findings at the protein level, we performed immunofluorescence staining for *MYC* and *SRM* in patient-derived tissue specimens from breast and colorectal cancer (CRC) patients (Fig. 6C). To ensure clinical relevance, we specifically obtained tissue sections from patients with confirmed chemotherapy resistance and metastatic disease, comparing them to samples from chemosensitive, non-metastatic patients. Notably, our analysis validated significantly higher expression of both *MYC* and *SRM* in resistant metastatic tumors compared to non-metastatic cases in both breast (Fig. 6D–E) and colorectal cancer (Fig. 6F–G). These findings underscore the strong association between *MYC* and *SRM* in driving chemoresistance and metastatic progression.

### Machine learning-based classification identifies optimal cellular models for functional analysis of chemoresistance

To facilitate functional studies of chemoresistance and establish a resource for model selection, we developed a machine learning framework to classify cancer cell lines according to their chemoresistance state. Using transcriptomic profiles from the Cancer Cell Line Encyclopedia (CCLE), we calculated resistant and sensitive signature scores based on the 100-gene chemoresistance signature identified from our integrated single-cell analysis (Additional file 2: Table S4). These features were subsequently used to classify cell lines as resistant-like or sensitive-like (Additional file 1: Fig. 2B-C).

We evaluated multiple supervised machine learning algorithms, including Decision Tree, Logistic Regression, Support Vector Machine (SVM), and Multi-Layer Perceptron (MLP) models using an 80% training and 20% testing strategy. All models demonstrated high classification performance, with accuracies ranging from 96.4% to 100%, indicating robust discrimination between resistant-like and sensitive-like cellular states (Additional file 1: Fig. S2D). Application of this framework to cell lines derived from breast, lung, ovarian, and pancreatic cancers identified distinct resistant-like and sensitive-like populations (Additional file 1: Fig. S2C). Among these, MCF7 (breast cancer), PATU8988T (pancreatic cancer), and A2780/CP70 (ovarian cancer) were selected as representative resistant-like models for downstream functional studies.

To further validate their resistance profiles, we analysed their sensitivity to paclitaxel and cisplatin chemotherapy using data from the Genomics of Drug Sensitivity in Cancer (GDSC) within the DepMap database. As expected, PATU8988T, A2780\CP70 and MCF7 exhibited low sensitivity to paclitaxel and cisplatin, confirming their chemoresistant characteristics (Additional file 1: Fig. S2E). This is in line with earlier observation where studies showed low sensitivities to platinum and taxane-based therapies against MCF7 [51–53], A2780\CP70 [54, 55] and PATU8988T [56, 57] cells. These observations validate that our Atlas of cancer cell lines provides a robust resource for the community for a functional analysis.

### Therapeutic regime inhibiting *SRM, MYC*, and *WNT* activity enhances chemotherapy efficacy and overcomes resistance

**Fig. 7:**
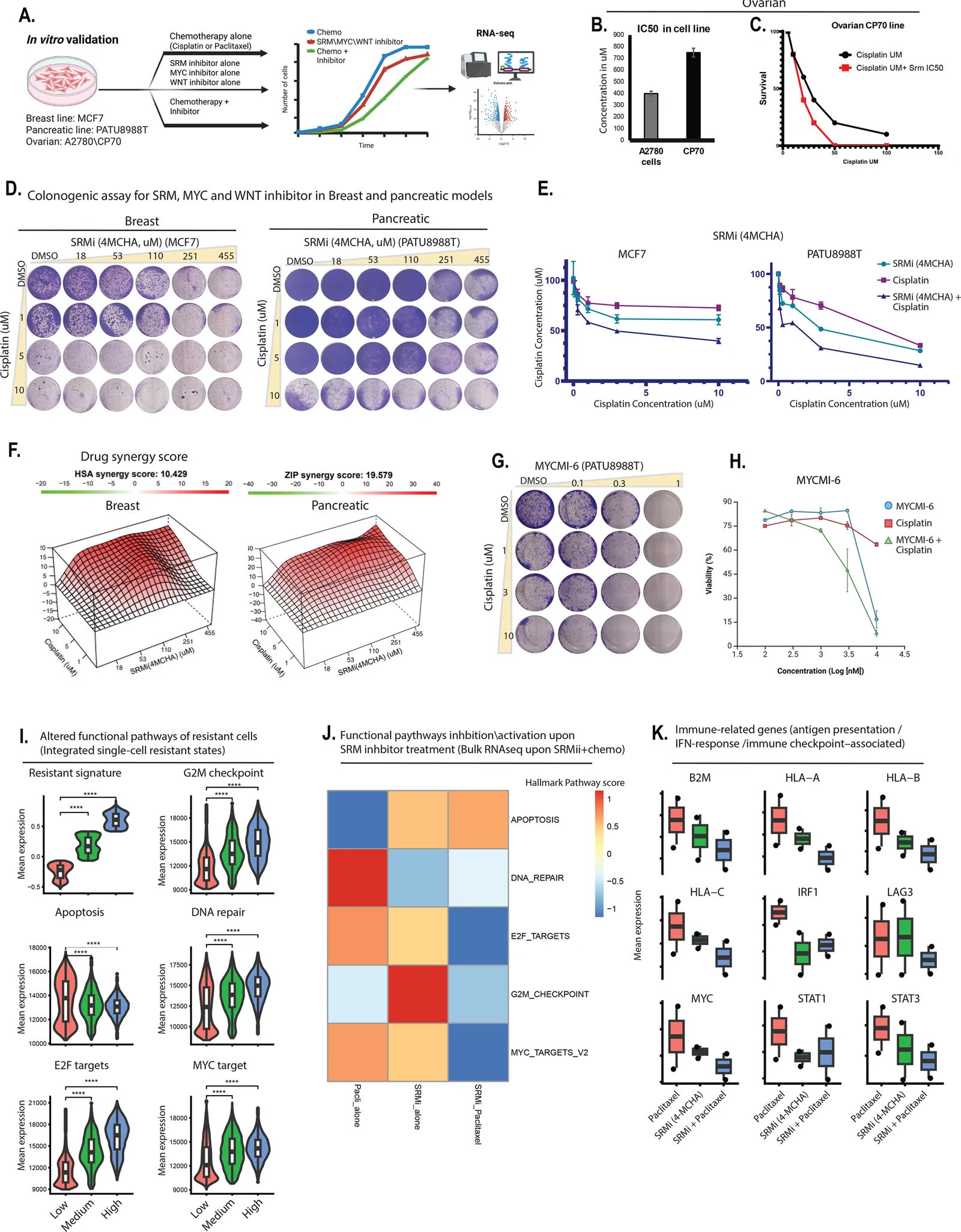
In vitro pharmacologic inhibition of SRM, MYC, and WNT reverses chemoresistance. **A.** Experimental workflow for in vitro (2D) validation combining chemotherapy with *SRM/MYC* inhibitors. **B.** Bar graph shows IC_50_ values of *SRM* inhibitor (4MCHA) across ovarian cancer cell lines. **C.** Cisplatin dose–response curves in A2780/CP70 cells with or without SRM inhibition. **D.** Colony formation assays of breast (MCF7) and pancreatic (PATU8988T) cancer cells treated with cisplatin and increasing concentrations of the SRM inhibitor 4-MCHA. **E.** Dose–response curves of cisplatin treatment with SRM inhibitor (4-MCHA) in MCF7 and PATU8988T cells. *SRM* inhibitor treatment was performed in 5 different doses (18, 53, 110, 251 and 455 uM). **F.** Drug synergy analysis for SRM inhibitor (4-MCHA) and cisplatin in breast and pancreatic models using HSA and ZIP synergy scores. **G.** Colony formation assays in PATU8988T cells treated with *MYC* inhibitor (MYCMI-6) and cisplatin. **H.** Dose–response curves of *MYC* inhibitor (MYCMI-6), cisplatin, and combination treatment. MycMI-6/Niclosamide treatments were performed in 5 different doses (0.1, 0.3, 1, 3 and 10 uM). **I.** Activity of resistance-associated functional pathways across low-, medium-, and high-resistance states in the integrated single-cell dataset. Statistical comparisons were performed using the Wilcoxon rank-sum test, with significance levels indicated on the plots. **J.** Hallmark pathway and custom signature activity across treatment groups. Heatmap showing mean ssGSEA scores for apoptosis, E2F targets, MYC targets, DNA repair, G2M checkpoint pathways, across Pacli_alone, SRMi_alone, and SRMi_Paclitaxel treatment groups. Scores were calculated for individual samples and averaged within each treatment group. Values are displayed as row-scaled pathway activity scores. **K.** Expression of immune-related genes associated with antigen presentation, interferon signaling, and immune checkpoint regulation across treatment conditions following SRM inhibition and chemotherapy

Given our findings that elevated *SRM* levels, high *MYC* activity, and enhanced *WNT* signaling are hallmarks of a high resistant state in chemoresistant tumors, we next investigated whether their inhibition could enhance the efficacy of chemotherapy in resistant cancer cells. To test therapeutic vulnerabilities, we evaluated the effects of specific inhibitors of *SRM* (SRMi, 4MCHA [58]), *MYC* (MYCMI-6 [59]), and WNT (Niclosamide [60]) alone and in combination with cisplatin or paclitaxel. We performed clonogenic growth and MTT assays using these inhibitors alone, chemotherapy alone, and in combination across three chemoresistant cell lines representing three cancer types: PATU8988T (pancreatic), Cp70 (ovarian), and MCF7 (breast) (Fig. 7A).

For the SRM inhibition experiment, we first confirmed that combining SRM inhibition with chemotherapy exerts a strong synergistic anti-cancer effect. To demonstrate this, we utilized two ovarian cancer cell lines: Cp70, a well-established cisplatin-resistant line [55], and its chemosensitive parental line A2780. The resistant status of Cp70 was validated by its higher IC_50_ for cisplatin compared to A2780 (Fig. 7B). Upon treatment with SRMi (4MCHA) (IC_50_) in combination with cisplatin at varying concentrations, A2780 cells showed enhanced inhibition of viability (Additional file 1: Fig. S3A). Notably, while Cp70 cells displayed resistance to cisplatin, the addition of SRMi (IC_50_) led to a complete inhibition of cell survival, demonstrating the potent combinatorial therapeutic potential of SRM inhibition in chemoresistant cells (Fig. 7C).

In the pancreatic cancer model (PATU8988T), combining SRMi (4MCHA) with low-dose cisplatin significantly enhanced anti-cancer activity in a synergistic manner (synergy score = 19.5), leading to approximately 70–80% inhibition of cell proliferation at the highest doses compared to single-agent treatments (Fig. 7D-F). A strong synergistic effect (synergy score = 10.4) was also observed when 4MCHA was combined with cisplatin in the breast cancer cell line MCF7 (Fig. 7D-F ). We observed a similar synergistic effect when combining SRMi with paclitaxel in both PATU8988T (Synergy score = 28.2) and MCF7 (Synergy score = 16.74) lines (Additional file 1: Fig. S3B-C, F).

To further evaluate the combinatorial effect of SRM inhibition with an additional chemotherapeutic agent, we performed clonogenic survival and MTT assays using SRMi alone, cisplatin or paclitaxel alone, and their combination. This combination demonstrated synergistic effects in both PATU8988T and MCF7 cells (Fig. 7F, Additional file 1: Fig. S3D-E, F), reinforcing the ability of SRM inhibition to enhance chemotherapy efficacy in resistant cancer models.

For the MYC inhibition experiment, we treated the cells with MYC inhibitor (MYCMI-6) alone and in combination with cisplatin in both breast and pancreatic lines. Treatment with MYCMI-6, a selective MYC inhibitor, showed potent synergy with cisplatin (Fig. 7G, H, Additional file 1: Fig. S3G). The combination of MYCMI-6 and cisplatin led to significantly greater reductions in colony formation compared to either drug alone in both breast and pancreatic models. These results functionally validate the MYC–SRM axis in driving chemotherapy resistance. Given the association of *WNT* signaling with stemness and survival, we tested Niclosamide, a *WNT* pathway inhibitor. Similar to *SRM* and MYCMI-6, Niclosamide in combination with cisplatin significantly reduced colony survival in MCF7 and PATU8988T cells (Additional file 1: Fig. S3H), highlighting the role of *WNT* signaling as another resistance-supporting pathway. Dose-response viability assays confirmed that *SRM* inhibition + cisplatin yielded superior efficacy over monotherapies across cell lines (Additional file 1: Fig. S3I). Furthermore, qPCR results indicate a substantial inhibition of *SRM* mRNA expression upon treatment with the MYC inhibitor (MYCMI-6) and its further reduction when it is combined with cisplatin in both MCF7 and PATU8988T cells (Additional file 1: Fig. S3J).

To mechanistically connect SRM targeting with transcriptional programs underlying chemoresistance, we first examined pathway activities associated with resistant cell states identified in our integrated single-cell analysis (Fig. 2). As shown in Fig. 7I, chemoresistant states were characterized by enrichment of cell cycle checkpoints, DNA repair, MYC targets, and E2F signaling, while apoptosis was comparatively reduced, indicating a survival advantage and impaired cell death programs in high-resistance states. Based on these observations, we next assessed whether these pathways are modulated upon SRM inhibition in combination with chemotherapy. Bulk RNA-seq analysis revealed that SRM inhibitor treatment, particularly in combination with chemotherapy (Paclitaxel), led to suppression of DNA damage response and checkpoint-associated pathways, alongside reactivation of apoptotic programs and modulation of MYC/E2F transcriptional activity (Fig. 7J). Notably, the combination treatment induced apoptosis relative to chemotherapy alone, consistent with increased cell death and enhanced therapeutic response. We further used transcriptomic profiling data from pancreatic cancer cells subjected to Paclitaxel, SRM inhibitor, and combination (Paclitaxel + SRM inhibitor) treatments to assess the impact on immune regulatory pathways. The combination therapy led to downregulation of immune checkpoint and antigen presentation genes (*B2M, HLA A/B/C, IRF1, STAT1, STAT3*), consistent with suppression of interferon response and MYC-associated immune escape program (Fig. 7K). Together, these findings demonstrate that SRM inhibition functionally reverses key transcriptional features of chemoresistance by suppressing proliferative and repair pathways while restoring apoptotic signaling.

The differential expression analysis between paclitaxel alone vs combination therapy with SRMi confirmed that SRM inhibition (4MCHA), especially in combination with paclitaxel, led to marked downregulation of the chemoresistant transcriptional program (Additional file 1: Fig. S3K). Pseudotime analysis of resistant state cells showed that genes downregulated in the SRM–Paclitaxel group were highly expressed in the high-resistant end of the trajectory, whereas upregulated genes were associated with less resistant phenotypes (Additional file 1: Fig. S3L), reinforcing the role of SRM targeting in reversing resistant transcriptional states.

Overall, these findings establish that pharmacological targeting of SRM and MYC reprograms resistant tumor cells, restores chemotherapy sensitivity, and reverses the molecular phenotype of resistance. This provides compelling evidence for the SRM-MYC axis as a therapeutic vulnerability in drug-refractory cancers.

### SRM-driven chemoresistance in patient-derived organoids and therapeutic reversal with SRM inhibition

**Fig. 8:**
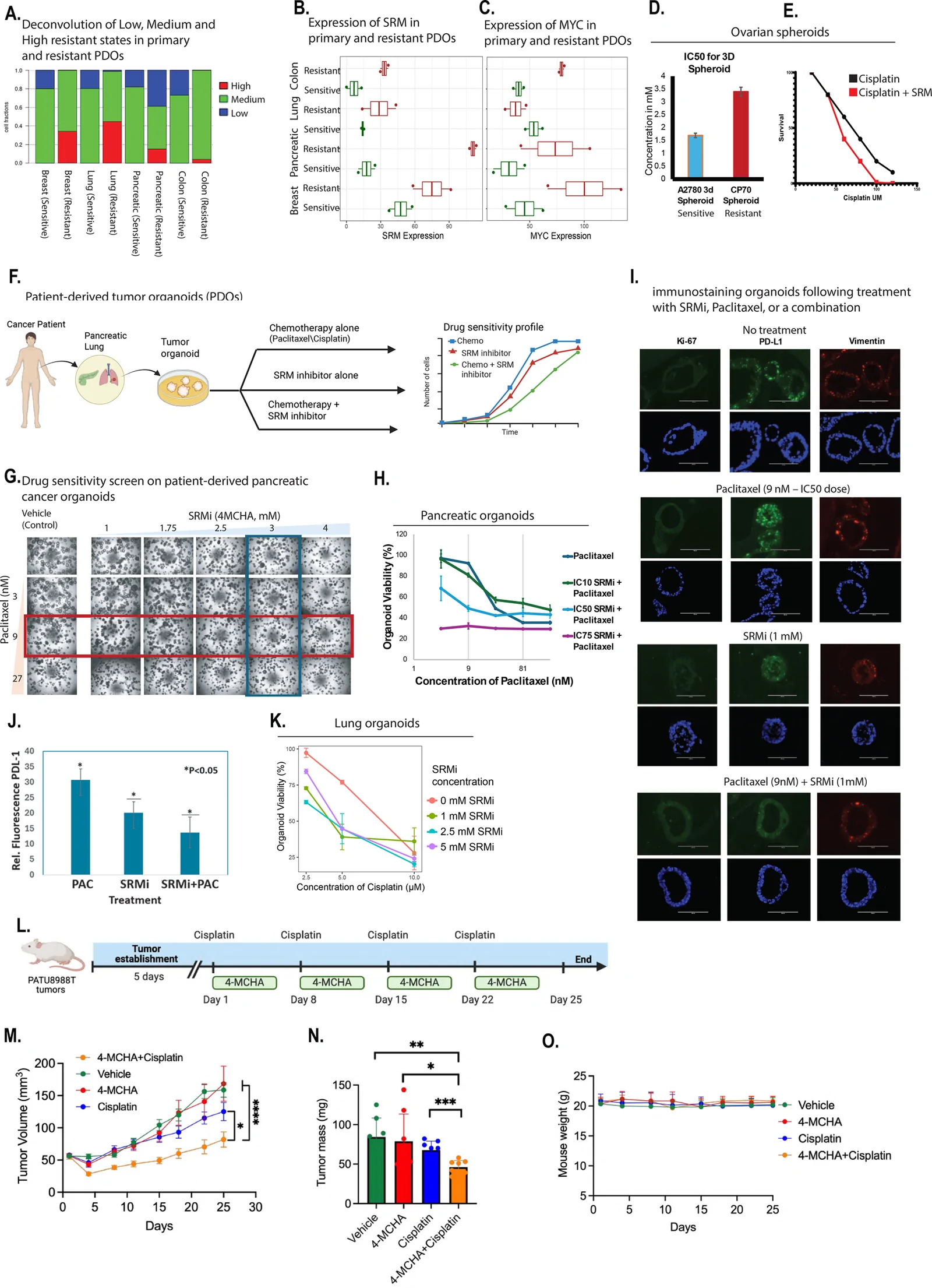
SRM inhibition re-sensitizes PDOs to chemotherapy. **A.** Deconvolution of low, medium, and high resistant states in sensitive and resistant patient-derived organoids (PDOs) across multiple cancer types. **B.** Boxplots showing expression of SRM across resistant and sensitive organoid types. **C.** Boxplot of MYC expression across PDO models. **D.** Cisplatin IC50 values across spheroids showing higher resistance in SRM-high lines. **E.** Cisplatin dose–response curves in ovarian spheroids treated with increasing concentrations of the SRM inhibitor 4-MCHA. **F.** Experimental workflow for generation, treatment, and drug sensitivity testing of patient-derived tumor organoids. **G.** Brightfield images of pancreatic spheroids treated with paclitaxel and increasing concentrations of the SRM inhibitor 4-MCHA. **H.** Viability of pancreatic PDOs following treatment with paclitaxel alone or in combination with increasing concentrations of 4-MCHA. **I.** Immunofluorescence staining of patient-derived pancreatic tumor organoids treated with vehicle control, paclitaxel (9 nM; IC_50_), SRM inhibitor (SRMi, 1 mM), or combination therapy. Organoids were stained for *Ki-67* (proliferation), *PD-L1* (immune checkpoint), and Vimentin (EMT marker). Scale bar in all images = 100 uM. **J.** Quantification of *PD-L1* expression following treatment with paclitaxel, *SRM* inhibitor, or combination therapy. **K.** Growth curve shows effects of chemotherapy, SRMi alone and in combination in lung cancer organoids. The lung cancer organoids were derived from NCI and treated with SRMi, cisplatin and combination therapy. **L.** In vivo experimental design for xenograft studies, including treatment schedule for cisplatin and SRM inhibitor (4-MCHA). **M.** Growth of PATU8988T tumors in female NOG *mice* when treated with vehicle (*n* = 8), cisplatin (5 mg/kg, *n* = 8), 4-MCHA (5 mg/kg, *n* = 8) or combined cisplatin and 4-MCHA (*n* = 8). Tumor growth curves under different treatment conditions (vehicle, SRM inhibitor, cisplatin, and combination), demonstrating reduced tumor burden with combination therapy. **N.** Endpoint tumor mass measurements from PATU8988T xenografts following treatment. **O.***Mouse* body weight over the course of treatment. Statistical difference was determined by the two-way ANOVA method followed by following Bonferoni’s multiple correction testing or by student’s t-test. *0.05 > *p* ≥ 0.01, **0.01 > *p* ≥ 0.001, ***0.001 > *P.* NS non-significant

Patient-derived tumor organoids (PDOs) have emerged as powerful preclinical models that faithfully recapitulate the histological, molecular, and genetic characteristics of the original tumors, enabling physiologically relevant studies of tumor biology and therapeutic response [61, 62]. Here we obtained PDOs from chemotherapy responsive and resistant patients across multiple cancer types, including breast, lung, pancreatic, and colon cancers and performed deconvolution analysis to assess the presence of chemoresistant states. Interestingly, all PDOs contained measurable proportions of low, medium, and high resistance states, with chemoresistant samples enriched for high-resistance cells as compared to the chemosensitive ones (Fig. 8A). This underscores that resistance programs defined in bulk and single-cell analyses are also maintained in ex vivo 3D tumor models.

In line with these observations, SRM was expressed at higher levels in PDOs from chemo resistant patients (Fig. 8B). These findings align with earlier observations in tumors and cell lines (Figs. 6 and 7), supporting the utility of SRM as a marker of high-resistance burden in PDOs. Furthermore, consistent with the MYC–SRM axis established in previous figures, MYC expression was also significantly elevated in chemoresistant PDOs, with expression trends paralleling those of SRM (Fig. 8C). These results suggest that the chemoresistant subpopulation in patient tumors is transcriptionally coordinated by MYC and sustained by SRM activity.

We next extended our therapeutic exploration to 3D-spheroid and PDOs. Drug sensitivity profiling in 3D-spheroid models confirmed that cisplatin-resistant spheroid (e.g., CP70) had higher IC_50_ values for cisplatin compared to sensitive lines like A2780 (Fig. 8D). Notably, co-treatment with SRM inhibitor (4MCHA) significantly lowered cisplatin IC_50_ in resistant Cp70 spheroids (Fig. 8E), demonstrating restored chemosensitivity.

Furthermore, PDOs generated from pancreatic and lung cancer patients were treated with paclitaxel alone, or in combination with SRM inhibitor (Fig. 8F). Drug response assays showed that SRM inhibitor monotherapy partially reduced pancreatic organoid viability, while combination treatment with chemotherapy produced the strongest and most sustained suppression of tumor growth (Fig. 8G). Microscopy images of pancreatic PDOs demonstrated visually reduced size and density of organoids treated with SRMi + paclitaxel, compared to either agent alone. Quantitative viability assays (Fig. 8H) confirmed a dose-dependent decrease in survival across multiple organoids treated with the combination regimen, further validating the broad and translational impact of SRM targeting in resistant tumor models. To assess whether SRM inhibition alters key resistance-associated phenotypes, we performed immunofluorescence staining of pancreatic cancer organoids treated with paclitaxel, SRMi, or combination therapy. We focused on three clinically relevant markers: *Ki-67* (proliferation), *PD-L1* (immune evasion), and Vimentin (epithelial–mesenchymal transition). Compared to untreated controls, paclitaxel treatment led to a visible increase in the expression of all three markers, indicative of enhanced proliferative capacity, immune escape, and phenotypic plasticity, hallmarks of therapy resistance (Fig. 8I–J). In contrast, SRMi monotherapy or its combination with paclitaxel resulted in reduced expression of *PD-L1* and Vimentin, suggesting that SRM inhibition may suppress resistance-associated signaling and mitigate tumor plasticity (Fig. 8I–J). These observations support the proposed role of *SRM* as a driver of chemoresistant tumor states. A similar effect was observed in lung cancer organoids, where combining SRM inhibitor with chemotherapy dramatically improved chemotherapy response (Fig. 8K).

We next assessed the vivo efficacy of cisplatin (5 mg/kg) and SRM inhibitor 4-MCHA (5 mg/kg), administered individually or in combination, in *mice* bearing PATU8988T xenograft tumours (Fig. 8L). 4-MCHA was delivered orally five days per week, while cisplatin was given intraperitoneally once weekly. Neither 4-MCHA nor cisplatin alone produced a significant reduction in tumour growth compared with vehicle controls. In contrast, combined treatment with 4-MCHA and cisplatin markedly suppressed tumor growth, resulting in significantly smaller tumor volumes and masses than either monotherapy or control (Fig. 8M–N). Importantly, no significant changes in body weight were observed across treatment groups throughout the four-week study period, and no behavioral or visible adverse effects were detected, indicating good systemic tolerability of SRM inhibition in vivo (Fig. 8O).

Collectively, these findings confirm that the chemoresistance transcriptional state, defined by MYC activation and SRM expression, is conserved in PDOs across cancer types and is functionally targetable using SRM inhibition. SRMi restores chemotherapy sensitivity in resistant PDOs, providing a strong preclinical rationale for combination therapies that suppress the MYC–SRM resistance axis in aggressive cancer patients.

## Discussion

Chemoresistance remains a major obstacle in the treatment of solid tumors and is driven by both intrinsic tumor heterogeneity and adaptive responses to therapy. While resistance has traditionally been associated with epithelial–mesenchymal transition, stemness, or cellular dormancy, our findings support an alternative model in which resistant tumor cells retain high proliferative capacity and actively engage transcriptional and metabolic programs that promote survival under therapeutic stress [63, 64].

By integrating single-cell transcriptomics, spatial transcriptomics, regulatory network analysis, and functional validation across multiple cancer types, we identify a conserved chemoresistant state characterized by elevated G2/M and S-phase cycling, activation of MYC and E2F transcriptional programs, enhanced DNA repair, and increased PI3K/AKT signaling. These observations are consistent with emerging evidence demonstrating that resistant tumor populations maintain proliferative fitness and exploit checkpoint and DNA-repair pathways to survive cytotoxic therapies [65, 66]. At the regulatory level, our analyses identify MYC as a central orchestrator of the resistance program. Pseudotime and gene regulatory network analyses revealed progressive MYC activation during resistance acquisition, while spatial and bulk transcriptomic analyses demonstrated enrichment of MYC-associated programs within resistant epithelial tumor compartments. These findings are consistent with previous studies showing that MYC promotes resistance through metabolic rewiring, DNA-repair activation, and suppression of apoptosis [67–72].

A key and novel finding of this study is the identification of SRM (Spermidine Synthase) as a pan-cancer marker and functional effector of the chemoresistant state. Spermidine Synthase encodes the enzyme responsible for converting putrescine into spermidine and plays a pivotal role in polyamine metabolism, because of their direct involvement in cellular processes frequently dysregulated in tumors [58, 73]. Polyamine metabolism is widely implicated in cancer-cell proliferation, survival, and therapeutic response [74–79]. We show a preferential MYC occupancy at the *SRM* locus in resistant-like compared with sensitive-like cells, while functional enrichment of MYC-bound genes highlights pathways critical to DNA repair and mitosis. Together with the reduced *SRM* expression following pharmacological MYC inhibition, these findings support MYC-mediated regulation of *SRM* as one component of the broader chemoresistance program. Our findings complement earlier studies identifying polyamine metabolism as a MYC-driven vulnerability in multiple cancers [80–83].

Notably, although global MYC chromatin occupancy was generally increased in resistant-like cell lines, *SRM* expression did not scale proportionally with either the total number of MYC-binding sites or chemotherapy resistance across individual models. This likely reflects the distinction between global MYC occupancy, which represents a broad transcriptional state, and steady-state SRM expression, which may additionally be influenced by cell-context-dependent regulatory mechanisms. Thus, our findings do not support SRM expression as a universal standalone quantitative predictor of drug resistance. Rather, preferential MYC occupancy at the *SRM* locus in resistant-like cells, suppression of *SRM* expression following MYC inhibition, and functional re-sensitization upon SRM inhibition collectively support a context-dependent MYC–SRM regulatory axis that contributes to the chemoresistant state.

Beyond tumor-intrinsic effects, our data suggest that the MYC–SRM axis may contribute to immune evasion. SRM inhibition reduced *PD-L1* expression and suppressed antigen-presentation and interferon-response pathways in pancreatic organoid models, suggesting that polyamine metabolism may couple chemoresistance with immune-regulatory programs. Further studies in immune-competent models will be required to define the therapeutic implications of these observations. The translational potential of this axis is supported by cancer cell lines, 3D patient-derived organoid (PDO) and xenograft models. Pharmacological inhibition of SRM, MYC (using MYCMI-6), or the WNT pathway (via niclosamide) significantly reversed chemoresistance across tumor models and PDOs. Targeting SRM with a pharmacologic inhibitor (4MCHA), alone or in combination with cisplatin or paclitaxel, significantly reverses chemoresistance across breast, ovarian, and pancreatic models. Furthermore, inhibitors of MYC (MYCMI-6) and WNT (Niclosamide) recapitulate these effects, further emphasizing the convergence of resistance maintenance on a MYC–WNT–SRM module. Drug-treated cells showed consistent suppression of MYC targets, E2F signaling, and DNA repair genes, highlighting a convergence of survival circuits on the MYC–SRM axis. Transcriptomic analyses post-treatment show downregulation of hallmark resistance pathways i.e. DNA repair, E2F, MYC signaling, and upregulation of apoptotic programs, confirming re-sensitization. In vivo validation using a PATU8988T xenograft model demonstrated that combined SRM inhibition (4‑MCHA) and cisplatin produced a synergistic suppression of tumor growth and mass compared with either monotherapy, without measurable toxicity. These findings provide strong preclinical proof‑of‑concept that SRM targeting enhances chemotherapy efficacy while remaining well‑tolerated in vivo. These results also validate previous reports where MYC-targeted therapies restored sensitivity in treatment-refractory models [84, 85].

Notably, MYC inhibition has garnered significant interest in recent years and has progressed to clinical trials for the treatment of various cancers [86]. In colorectal cancer, MYC collaborates with SMS (another key gene in the polyamine metabolism pathway) to sustain cell survival by suppressing the pro-apoptotic protein Bim, and co-inhibition of SMS and MYC has been shown to reduce tumor burden [81]. In line with these findings, our study demonstrates the cooperative interaction and spatial co-localization of MYC with SRM in chemoresistant cells. Based on this, we propose that dual inhibition of MYC and SRM could effectively suppress tumor growth across multiple cancer types, offering a promising therapeutic strategy for aggressive, treatment-resistant malignancies.

Furthermore, the clinical relevance of our findings is reinforced by survival analyses across large patient cohorts, where high-resistance signatures predict poor outcomes in breast, lung, and ovarian cancer. These clinical correlations, combined with the PDO re-sensitization data, underscore the robustness and clinical applicability of targeting the MYC–SRM axis. Importantly however, although SRM inhibition re-sensitized PDOs and cell lines, the systemic tolerability and pharmacokinetics of SRM inhibitors require further study. Finally, while MYC is a central hub, resistance likely involves combinatorial regulation by additional factors, including *FOXM1*, *HSF1*, and *NRF2*, whose mechanistic contributions deserve deeper investigation [88, 89].

Spatial transcriptomic profiling revealed that resistant cells cluster within epithelial tumor compartments, indicating localized niche protection rather than broad EMT activation. This refines current paradigms and suggests that resistance programs are spatially and functionally constrained within proliferative epithelial domains rather than diffusely across stromal or inflammatory components [90].

While prior studies have implicated MYC‑driven polyamine metabolism in cancer progression and therapy resistance within select tumor types, our work substantially extends these observations by establishing a pan‑cancer, systems‑level framework of chemoresistance. Through the integration of single‑cell, spatial, and bulk transcriptomic datasets across multiple malignancies, we define a conserved, therapy‑selected transcriptional state marked by progressive *MYC* activation and proliferative capacity. Within this state, SRM emerges as a functional downstream effector of MYC enriched across resistant tumors and spatially confined to malignant epithelial compartments. Comprehensive multi‑modal analyses—combining MYC ChIP‑seq, protein co‑localization, and functional perturbations, provide evidence supporting regulatory coupling between MYC and SRM in chemoresistant contexts. Functional studies across cell lines, PDOs, and in vivo models further establish that *SRM* inhibition reverses chemoresistance, underscoring its causal role rather than a correlative association.

## Conclusions

Our study defines a conserved pan-cancer chemoresistance program across multiple tumor types and identifies a highly resistant malignant cell state characterized by proliferative activity, enhanced DNA repair, suppressed apoptotic signaling, and increased MYC activity. We identify SRM as a key marker and functional vulnerability of this resistant state and establish a MYC–SRM regulatory axis associated with chemotherapy resistance. Pharmacological inhibition of SRM enhanced chemotherapy efficacy across cancer cell lines, PDOs, and an in vivo tumor model, supporting the translational potential of targeting this pathway. Collectively, these findings provide a framework for identifying chemoresistant tumor states across cancers and nominate SRM as a potential therapeutic target for overcoming treatment resistance.

## Electronic supplementary material

Below is the link to the electronic supplementary material.

Supplementary Material 1: Additional file 1: Supplementary Figures. Contains Figs. S1–S3 and their corresponding figure legends, providing additional validation of the pan-cancer chemoresistance signature and SRM expression, spatial transcriptomic and machine-learning analyses, and functional analyses of SRM inhibition in combination with chemotherapy

Supplementary Material 2: Additional file 2: Supplementary Tables. Contains Tables S1–S8, including clinical and treatment-response details of the single-cell transcriptomic samples, differentially expressed genes associated with chemoresistance, the pan-cancer chemoresistance gene signature, and additional gene signatures used in the study

## Data Availability

The single-cell RNA-sequencing datasets analyzed in this study are publicly available from the respective repositories. The breast cancer datasets are available through NCBI Gene Expression Omnibus (GEO) under accession GSE117309 [91] and NCBI BioProject under accession PRJNA396019 [92]. The small-cell lung cancer dataset is available through NCBI GEO under accession GSE138267 [93]. The ovarian cancer dataset is available through NCBI GEO under accession GSE165897 [94]. The pancreatic cancer datasets are available through NCBI GEO under accessions GSE205013 [95] and GSE202051 [96]. The spatial transcriptomic breast cancer dataset is available through NCBI GEO under accession GSE210616 [97], the ovarian cancer spatial transcriptomic dataset under GSE189843 [98], and the pancreatic cancer spatial and sequencing data through the Human Tumor Atlas Network/dbGaP under accession phs002371.v1.p1 [99]. The lung cancer spatial transcriptomic dataset was obtained from the publicly available 10x Genomics Visium Human Lung Cancer dataset [100]. The source code and computational workflows used for the analyses presented in this study are publicly available through GitHub [101]. The version associated with this publication has been permanently archived in Zenodo (version 1.0.0; DOI: https://doi.org/10.5281/zenodo.22028670) [102]. Detailed sample-level information, including cancer type, treatment, response status, source study, and dataset accession, is provided in Additional file 2: Table S1. All single-cell transcriptomic datasets analyzed in this work can additionally be explored through the PANCANCER Resistome repository at: https://panresistome.shinyapps.io/PANRESISTOMEMAP/. Additional bulk RNAseq data generated and analyzed in the current study are available within Additional file 2: Table S8.
