# Supplementary material for: Uncovering pan-cancer signatures of chemoresistance
Source: Genome Med. 2026 Sep 16;18:141. doi: 10.1186/s13073-026-01763-2 (PMC13625386; doi:10.1186/s13073-026-01763-2)
Supplement: Supplementary file 1 [file 13073_2026_1763_MOESM1_ESM.pdf]

Additional files

Figure S1

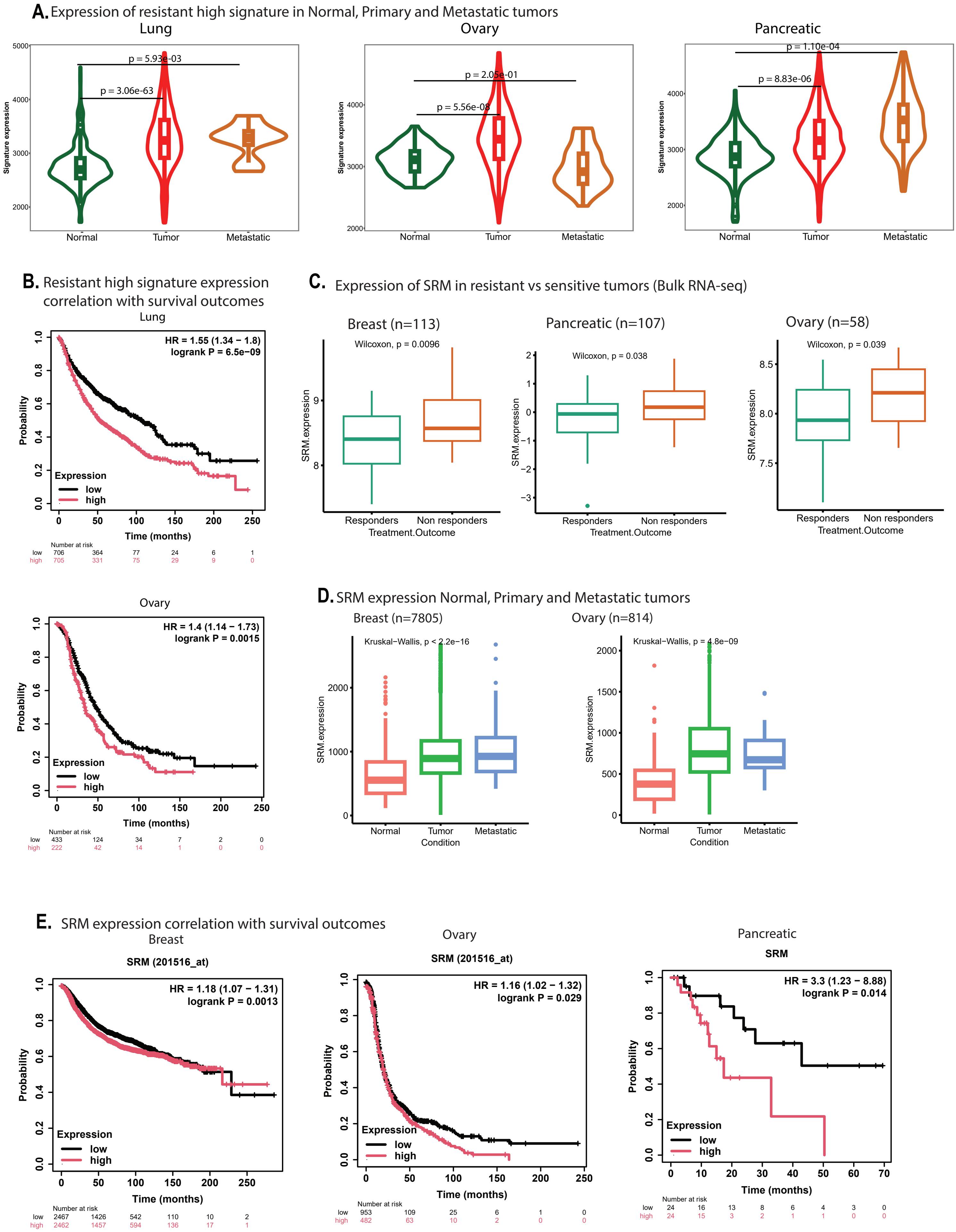
A. Expression of resistant high signature in Normal, Primary and Metastatic tumors
Lung
Ovary
Pancreatic
Signature expression
p = 5.93e-03
p = 3.06e-63
p = 2.05e-01
p = 5.56e-08
p = 1.10e-04
p = 8.83e-06
Normal
Tumor
Metastatic
B. Resistant high signature expression correlation with survival outcomes
Lung
HR = 1.55 (1.34 − 1.8)
logrank P = 6.5e−09
Ovary
HR = 1.4 (1.14 − 1.73)
logrank P = 0.0015
Probability
Time (months)
Expression
low
high
Number at risk
low 706 364 77 24 6 1
high 705 331 75 29 9 0
low 433 124 34 7 2 0
high 222 42 14 1 0 0
C. Expression of SRM in resistant vs sensitive tumors (Bulk RNA-seq)
Breast (n=113)
Wilcoxon, p = 0.0096
Pancreatic (n=107)
Wilcoxon, p = 0.038
Ovary (n=58)
Wilcoxon, p = 0.039
SRM.expression
Responders
Non responders
Treatment.Outcome
D. SRM expression Normal, Primary and Metastatic tumors
Breast (n=7805)
Kruskal−Wallis, p < 2.2e−16
Ovary (n=814)
Kruskal−Wallis, p = 4.8e−09
SRM.expression
Normal
Tumor
Metastatic
Condition
E. SRM expression correlation with survival outcomes
Breast
SRM (201516_at)
HR = 1.18 (1.07 − 1.31)
logrank P = 0.0013
Ovary
SRM (201516_at)
HR = 1.16 (1.02 − 1.32)
logrank P = 0.029
Pancreatic
SRM
HR = 3.3 (1.23 − 8.88)
logrank P = 0.014
Probability
Time (months)
Expression
low
high
Number at risk
low 2467 1426 542 110 10 2
high 2462 1457 594 136 17 1
low 953 109 25 6 1 0
high 482 63 10 2 0 0
low 24 16 13 8 6 4 3 0
high 24 15 3 2 1 1 0 0

Fig. S1. SRM expression and clinical outcomes across tumor types.

A. Expression of the chemoresistance signature in normal, primary tumor, and metastatic samples from lung, ovarian, and pancreatic cancer cohorts. The group differences across Normal, Tumor, and Metastatic samples were assessed using the Kruskal–Wallis test followed by Dunn's post hoc multiple comparisons test, with adjusted p values reported.

B. Kaplan–Meier survival analysis of lung and ovarian cancer patients stratified by chemoresistance signature expression. Survival differences were evaluated using the log-rank (Mantel-Cox) test, with hazard ratios estimated using Cox proportional hazards models.

C. *SRM* expression in treatment responders and non-responders across breast, pancreatic, and ovarian cancer cohorts. The box-and-whisker plots, the horizontal lines mark the median, the box limits indicate the 25th and 75th percentiles, and the whiskers extend to 1.5× the interquartile range from the 25th and 75th percentiles. The statistical testing of expression levels of signature genes between the groups was performed using two-tailed unpaired Wilcoxon test.

D. Boxplots of *SRM* expression across conditions in large-scale TCGA breast (n=7805) and ovary (n=814) datasets. Group differences across Normal, Tumor, and Metastatic samples were assessed using a Kruskal–Wallis test, with p values shown on the plot.

E. Kaplan–Meier survival analysis of breast, ovarian, and pancreatic cancer patients stratified by SRM expression. Survival differences were evaluated using the log-rank (Mantel–Cox) test, with hazard ratios estimated using Cox proportional hazards models.

Figure S2

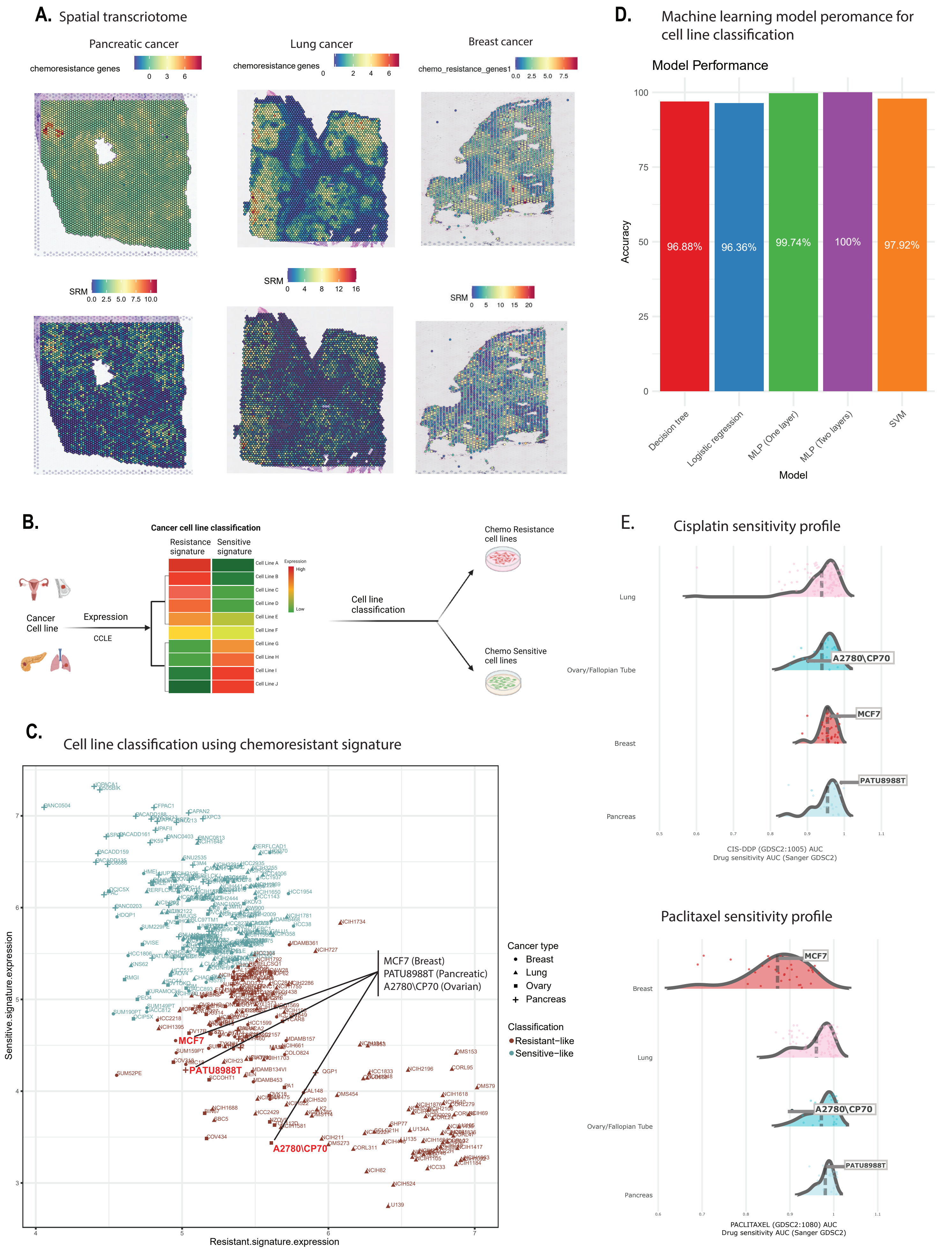

A. Spatial transcriotome
Pancreatic cancer
Lung cancer
Breast cancer
chemoresistance genes
chemo_resistance_genes1
SRM
D. Machine learning model peromance for cell line classification
Model Performance
Accuracy
Model
96.88%
96.36%
99.74%
100%
97.92%
Decision tree
Logistic regression
MLP (One layer)
MLP (Two layers)
SVM
B.
Cancer cell line classification
Resistance signature
Sensitive signature
Cancer Cell line
Expression
CCLE
Cell line classification
Chemo Resistance cell lines
Chemo Sensitive cell lines
E. Cisplatin sensitivity profile
Lung
Ovary/Fallopian Tube
Breast
Pancreas
A2780\CP70
MCF7
PATU8988T
CIS-DDP (GDSC2:1005) AUC
Drug sensitivity AUC (Sanger GDSC2)
C. Cell line classification using chemoresistant signature
Sensitive.signature.expression
Resistant.signature.expression
MCF7 (Breast)
PATU8988T (Pancreatic)
A2780\CP70 (Ovarian)
Cancer type
Breast
Lung
Ovary
Pancreas
Classification
Resistant−like
Sensitive−like
Paclitaxel sensitivity profile
PACLITAXEL (GDSC2:1080) AUC
Drug sensitivity AUC (Sanger GDSC2)

Fig. S2. Validation of SRM and chemoresistance gene expression in spatial and in vitro models.

A. Spatial transcriptomic heatmaps of chemoresistance signature and SRM in pancreatic, lung, and ovarian tumor samples.

B. Workflow for machine learning-based classification of cancer cell lines. Transcriptomic profiles from the Cancer Cell Line Encyclopedia (CCLE) were used to calculate chemoresistance and chemosensitivity signature scores. These features were used to train and evaluate multiple machine learning classifiers using an 80% training and 20% testing strategy, enabling prediction of resistant-like and sensitive-like cellular models.

C. Classification of cancer cell lines based on chemoresistance and chemosensitivity signature expression. Each point represents an individual cell line positioned according to its resistant-signature and sensitive-signature scores. Cell lines predicted as resistant-like or sensitive-like by the machine learning classifier are shown in different colors. Representative resistant-like cell lines selected for functional validation, including MCF7 (breast), PATU8988T (pancreatic), and A2780/CP70 (ovarian), are highlighted.

D. Performance of machine learning models for cell line classification. Model accuracy was evaluated using an 80% training and 20% testing strategy across multiple classifiers, including Decision Tree, Logistic Regression, Multi-Layer Perceptron (one-layer and two-layer), and Support Vector Machine (SVM). All models achieved high classification accuracy (>95%) in distinguishing resistant-like and sensitive-like cancer cell lines based on chemoresistance and chemosensitivity signature scores.

E. Drug sensitivity profiles for cisplatin and paclitaxel across cancer cell lines from the Genomics of Drug Sensitivity in Cancer (GDSC) database. Density distributions show area-under-the-curve (AUC) values across cancer types, with highlighted resistant-like cell lines exhibiting reduced drug sensitivity (higher AUC values).

Figure S3

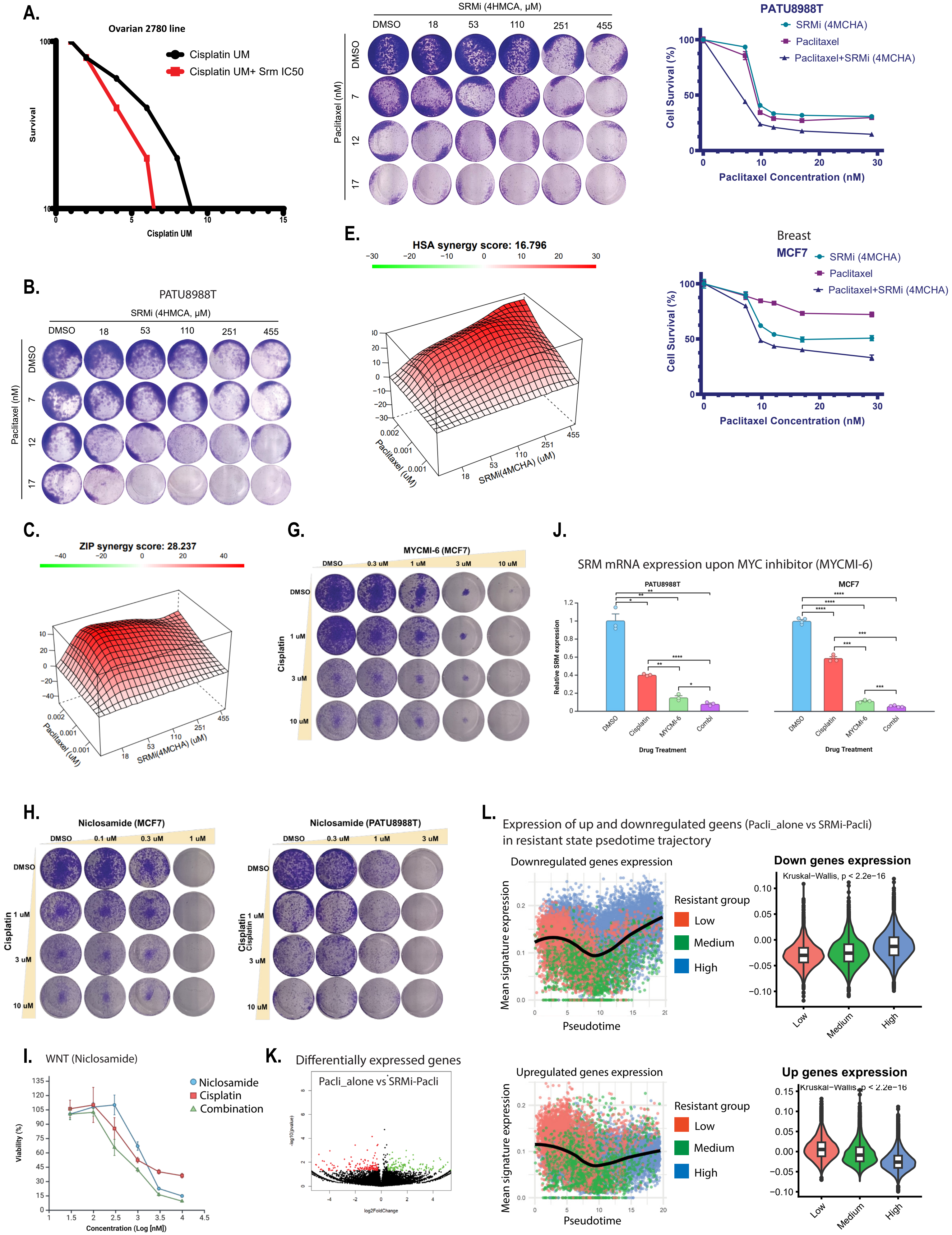

A.
Ovarian 2780 line
Cisplatin UM
Cisplatin UM+ Srm IC50
Survival
Cisplatin UM
D.
MCF7
SRMi (4HMCA, μM)
DMSO
18
53
110
251
455
Paclitaxel (nM)
DMSO
7
12
17
F.
Pancreatic
PATU8988T
SRMi (4MCHA)
Paclitaxel
Paclitaxel+SRMi (4MCHA)
Cell Survival (%)
Paclitaxel Concentration (nM)
Breast
MCF7
SRMi (4MCHA)
Paclitaxel
Paclitaxel+SRMi (4MCHA)
Cell Survival (%)
Paclitaxel Concentration (nM)
E.
HSA synergy score: 16.796
Paclitaxel (uM)
SRMi(4MCHA) (uM)
B.
PATU8988T
SRMi (4HMCA, μM)
DMSO
18
53
110
251
455
Paclitaxel (nM)
DMSO
7
12
17
C.
ZIP synergy score: 28.237
Paclitaxel (uM)
SRMi(4MCHA) (uM)
G.
MYCMI-6 (MCF7)
DMSO
0.3 uM
1 uM
3 uM
10 uM
Cisplatin
DMSO
1 uM
3 uM
10 uM
J.
SRM mRNA expression upon MYC inhibitor (MYCMI-6)
PATU8988T
MCF7
Relative SRM expression
DMSO
Cisplatin
MYCMI-6
Combi
Drug Treatment
H.
Niclosamide (MCF7)
DMSO
0.1 uM
0.3 uM
1 uM
Cisplatin
DMSO
1 uM
3 uM
10 uM
Niclosamide (PATU8988T)
DMSO
0.3 uM
1 uM
3 uM
Cisplatin
DMSO
1 uM
3 uM
10 uM
L.
Expression of up and downregulated geens (Pacli_alone vs SRMi-Pacli)
in resistant state psedotime trajectory
Downregulated genes expression
Mean signature expression
Pseudotime
Resistant group
Low
Medium
High
Down genes expression
Kruskal−Wallis, p < 2.2e−16
Low
Medium
High
I.
WNT (Niclosamide)
Niclosamide
Cisplatin
Combination
Viability (%)
Concentration (Log [nM])
K.
Differentially expressed genes
Pacli_alone vs SRMi-Pacli
log2FoldChange
Upregulated genes expression
Mean signature expression
Pseudotime
Resistant group
Low
Medium
High
Up genes expression
Kruskal−Wallis, p < 2.2e−16
Low
Medium
High

Fig. S3. Synergy analysis of SRM inhibition and chemotherapy in vitro.

A. Cisplatin dose–response curves in A2780 ovarian cancer cells treated with cisplatin alone or in combination with the *SRM* inhibitor 4-MCHA.

B. Colony formation assays of PATU8988T pancreatic cancer cells treated with paclitaxel and increasing concentrations of the *SRM* inhibitor 4-MCHA.

C. ZIP synergy analysis of combined *SRM* inhibitor (4-MCHA) and paclitaxel treatment in MCF7 breast cancer cells.

D. Colony formation assays of MCF7 breast cancer cells treated with paclitaxel and increasing concentrations of the *SRM* inhibitor 4-MCHA.

E. HSA synergy analysis of combined SRM inhibitor (4-MCHA) and paclitaxel treatment in MCF7 breast cancer cells.

F. Cell viability of PATU8988T and MCF7 cells following treatment with paclitaxel alone, SRM inhibitor (4-MCHA) alone, or combination therapy.

G. Colony formation assays in MCF7 cells treated with *MYC* inhibitor (MYCMI-6).

H. Colony formation assays in MCF7 and PATU8988T cells treated with WNT inhibitor (Niclosamide) and cisplatin.

I. Dose–response curves for WNT inhibitor (Niclosamide), cisplatin, and combination treatment.

J. Relative *SRM* mRNA expression levels upon treatment with DMSO, cisplatin, MYCMI-6 and combination treatments in MCF7 and PATU8988T cells. IC_{50} concentrations were used for cisplatin and MYCMI-6 treatments. Unpaired t-test was performed (*: <0.05, **: <0.01, ***: <0.001 and ****: <0.0001).

K. Volcano plot showing differentially expressed genes between paclitaxel alone and *SRM* inhibitor plus paclitaxel treatment.

L. Expression dynamics of genes upregulated and downregulated following *SRM* inhibitor plus paclitaxel treatment across the resistance pseudotime trajectory. Violin plots show signature activity across Low-, Medium-, and High-resistance states. Statistical significance was assessed using the Kruskal–Wallis test, with p values indicated on the plots.
